# Recommendations for Off-Label Drug Use in Ophthalmology in China: A Clinical Practice Guideline

**DOI:** 10.3389/fphar.2022.919688

**Published:** 2022-05-24

**Authors:** Guangyao Li, Ningli Wang, Yu Zhang, Wenbin Wei, Hai Lu, Suodi Zhai, Chao Zhang

**Affiliations:** ^1^ Department of Pharmacy, Beijing Tongren Hospital, Capital Medical University, Beijing, China; ^2^ Beijing Tongren Eye Center, Beijing Key Laboratory of Ophthalmology and Visual Science, Beijing Tongren Hospital, Capital Medical University, Beijing, China; ^3^ Department of Pharmacy, Tongji Medical College, Union Hospital, Huazhong University of Science and Technology, Wuhan, China; ^4^ Department of Pharmacy, Peking University Third Hospital, Beijing, China

**Keywords:** off-label drugs use, ophthalmology, GRADE, evidence-based guideline, rational drug use, recommendations (guidelines), GRADE-CERQual

## Abstract

Off-label drugs use is widespread in ophthalmology due to the delay in drug approvals and package inserts update. It has been found to vary among different medical institutions in China, leading to safety problems since inappropriate use. Guidance is urgently needed regarding how best to use the drugs for unapproved indications and routes of administration. We aimed to develop an evidence-based guideline to guide off-label drugs used in ophthalmology in China. The practice guideline was developed by the Hospital Pharmacy Professional Committee, Chinese Pharmaceutical Association, following the *WHO handbook for guideline development*. The guideline was initially registered in the International Practice Guidelines Registry Platform (IPGRP-2021CN096). The clinical questions included in the guideline were identified through a three-round Delphi vote. Databases search was performed in PubMed, Embase, the Cochrane Library, ClinicalTrials.gov, Chinese National Knowledge Infrastructure, and WanFang Database from their inception to 31 March 2021. Systematic reviews and meta-analyses for each clinical question were conducted individually to synthesize available scientific evidence. The Grading of Recommendations Assessment, Development, and Evaluation (GRADE) approach was used to assess the quality of evidence and grade the recommendations’ strengths. The multidisciplinary guideline groups were set up, including ophthalmologists, pharmacists, methodology experts, pharmacologists, pharmacoeconomists, and lawyers. The guideline identified 25 clinical questions included. A total of 32 systematic reviews, including 24 conducted by the systematic review group and eight high-relevance published within 2 years, were referred to address these questions. Finally, the guideline presented 32 recommendations addressing 25 clinical questions, involving five strong recommendations and 27 weak recommendations for the treatment of ocular fundus, corneal disease, glaucoma, and endophthalmitis. Current evidence from clinical studies supports the off-label drugs used in ophthalmology. We developed an evidence-based guideline using a rigorous multidisciplinary approach to guide these usages in route clinical practice.

## Introduction

Vision loss contributes to a substantial public health burden worldwide, causing significant economic impact, reducing the quality of life, impacting educational opportunities, and increasing the risk of premature death (Steinmetz et al., 2021). World Health Organization data show that at least 2.2 billion people have blindness and vision impairment globally, of whom one billion cases could have been prevented or yet to be addressed (World Health Organization, 2021). In China, the prevalence of blindness and visual impairment increased rapidly from 1990 to 2019 and reached 59.28 million patients in 2019. The leading causes of vision loss include cataracts, uncorrected refractive error, glaucoma, age-related macular degeneration, and diabetic retinopathy (Xu et al., 2020). Pharmacological approaches play essential roles in the prevention and treatment of these ocular diseases (Ron et al., 2019).

Off-label drugs refer to pharmaceutical drugs used for an unapproved indication or in an unapproved age group, dosage, or route of administration. Off-label drug use is not prohibited in the United States and the European Union, in which physicians could prescribe off-label drugs with the support of extensive scientific evidence (Mello et al., 2009; Lenk and Duttge, 2014; Kesselheim et al., 2019). China’s latest Physician Law also allows off-label drug use based on medical evidence (National People’s Congress of China, 2021). In the absence of better treatment, it is necessary to use off-label drugs in clinical practice (Pandit 2021; Rusz et al., 2021). Some off-label medications even are the only optional therapy for patients with some rare diseases (Fung et al., 2021). However, most off-label medications have not been rigorously evaluated, which may become a significant hidden danger to drug safety.

The use of drugs for unapproved indications and routes of administration in ophthalmology has been widespread due to the delay in drug approvals and package inserts update in China (Li et al., 2018; Yang et al., 2018; Zang et al., 2020; Zhai et al., 2020; Gao et al., 2021). Previous studies revealed that the rate of irrational use of off-label drugs in ophthalmology prescription in large teaching hospitals was high, ranging from 10.5% to 26.88% (Song et al., 2018; Sun et al., 2018). However, most off-label drug uses in ophthalmology are supported by different levels of evidence of drug efficacy. Han et al. found that of all off-label drug use identified in China, 13.5% of off-label drugs were approved by the US Food and Drug Administration, 56.8% of them complied with international guidelines or expert consensus (Han et al., 2017). Nevertheless, the quality of evidence for ophthalmic medication efficacy varies in different medical institutions. The evidence for off-label drug use in teaching hospitals in developed areas was significantly more robust than that in non-teaching hospitals in undeveloped areas (Mei et al., 2019).

At present, there are only scattered studies to systematically evaluate the efficacy and safety of ophthalmic drugs used in new unapproved indications or non-recommended routes. Furthermore, no guidelines or consensuses regarding off-label drug use in ophthalmology have been developed. Therefore, we aimed to investigate the medications used in ophthalmology that have not yet been approved in China. Additionally, this study aimed to develop Chinese evidence-based guideline for off-label drugs in ophthalmology to standardize the ophthalmic prescriptions and promote their rational use among different medical institutions.

## Methods

**FIGURE 1:**
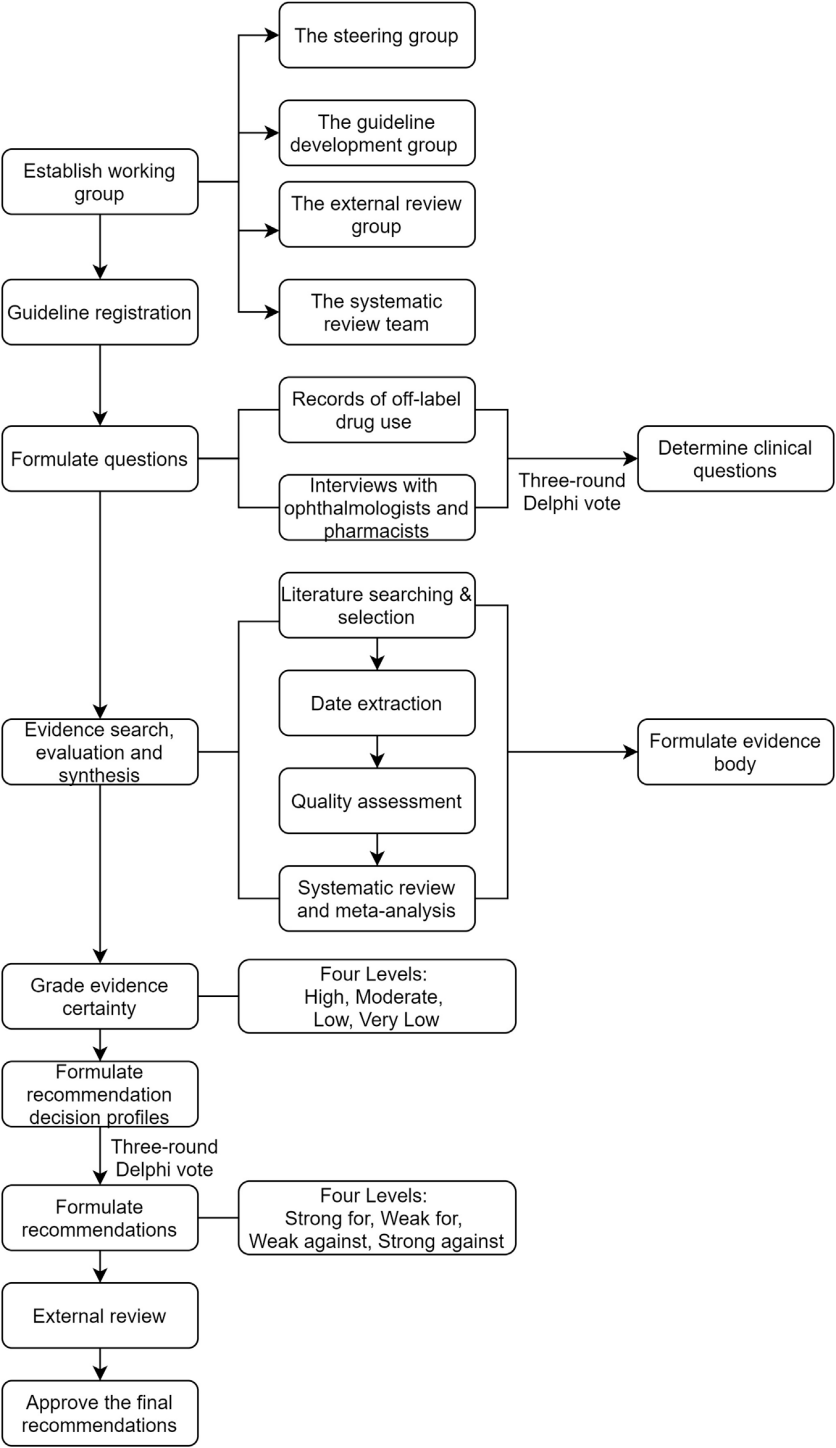
The flowchart of the guideline development process.

The guideline was developed following the WHO *Handbook for guideline development* and *the Appraisal of Guidelines for Research and Evaluation (AGREE II)* (Brouwers et al., 2010; World Health Organization, 2014). The guideline was drafted in accordance with the *Reporting Items for Practice Guidelines in Healthcare (The RIGHT)* statement (Chen et al., 2017). The flowchart of the guideline development process is presented in Figure 1.

### Sponsor and Registration

The project was led by the Hospital Pharmacy Professional Committee of the Chinese Pharmaceutical Society and implemented by the Department of Pharmacy, Beijing Tongren Hospital, Capital Medical University. Methodological support was obtained from the Chinese GRADE Center. The guideline was initially registered in the International Practice Guidelines Registry Platform (IPGRP-2021CN096).

### Guideline Scope

The guideline focused on off-label drugs used in ophthalmology in China, including medications in the treatment of ocular fundus, corneal disease, glaucoma, and endophthalmitis. Medications including antagonists of vascular endothelial growth factor (anti-VEGF) agents, immunosuppressive drugs, glucocorticoids, and antimicrobial agents were comprehensively evaluated to treat the above-mentioned disorders. Several drug usages approved in the United States and the European Union, not in China, were also included. The primary target populations were ophthalmologists, pharmacists, and nurses. Nevertheless, other health care professionals and policymakers involved in medication regulation can also benefit from this guideline.

### Guideline Working Group

According to the WHO *Handbook for guideline development*, the steering group, guideline development group (GDG), external review group, and systematic review (SR) group were established (Supplementary Table S1).

The steering group consisted of three clinical pharmacy experts (Chao Zhang, Yu Zhang, and Suodi Zhai), and three outstanding ophthalmologists (Ningli Wang, Wenbin Wei, and Hai Lu). The steering group’s role was to provide administrative support for the guideline development, make decisions at every stage, and control the entire process. Finally, they led the writing of the final draft of the guideline.

The GDG consisted of 21 multidisciplinary specialists: 11 clinical pharmacy specialists, five ophthalmology specialists, two methodology experts, one pharmacoeconomics consultant, one pharmacy-administration expert, and one health lawyer. They were responsible for determining the clinical questions, formulating recommendations, and reviewing and approving the final guideline document.

The SR group consisted of 16 qualified pharmacists or students trained in evidence-based medicine. They were responsible for collecting clinical questions, retrieving, evaluating, synthesizing, and grading evidence, conducting a systematic review, and formulating the summary of findings tables and the GRADE evidence profiles.

The external review group consisted of 29 first-line health care professionals (18 ophthalmologists and 11 pharmacists). They reviewed the final guideline to identify errors or missing data and commented on clarity, feasibility, setting-specific issues, and implementation implications.

### Formulation of Clinical Questions

In the early stage, the clinical questions about off-label drugs used in ophthalmology were collected from several hospitals by retrieval records and individual interviews with experienced ophthalmologists and pharmacists. Subsequently, a three-round Delphi vote was performed among the steering group and GDG to select the most important clinical questions that needed to answer. Finally, a total of 25 clinical questions were included in the guideline.

### Systematic Review

The systematic review was performed for each clinical question following the Patient, Intervention, Comparison, Outcome (PICO) framework in the *Cochrane Handbook for Systematic Reviews of Interventions* to answer the clinical questions selected (Higgins et al., 2021)*.* Databases including PubMed, Embase, the Cochrane Library, ClinicalTrials.gov, Chinese National Knowledge Infrastructure, and WanFang Database were comprehensively searched from their inception to 31 March 2021. Once one or more relevant, current, high-quality systematic reviews were found, these reviews were used when published or updated within 2 years.

The quality of systematic reviews/meta-analyses was assessed using the Assessment of Systematic Reviews (AMSTAR) tool (Shea et al., 2007), the quality of randomized controlled trials (RCTs) was determined using the Cochrane Collaboration’s tool (Sterne et al., 2019), the quality of cohort studies or case-control studies was assessed using the Newcastle-Ottawa Scale (NOS) (Stang, 2010), and the quality of case report or case series was evaluated using the Joanna Briggs Institute Critical Appraisal (JBI) tools (Moola et al., 2020). Disagreements were resolved by discussion or consultation with a third researcher. Meta-analyses of available studies were conducted using RevMan 5.4.

### Formulating Recommendations

The certainty of quantitative and qualitative evidence was assessed using the GRADE and GRADE-CERQual approaches, respectively (Guyatt et al., 2008b; Lewin et al., 2015). Furthermore, the strength of the recommendations was graded using the GRADE approach (Guyatt et al., 2008a; Guyatt et al., 2008b). For each clinical question, the SR group developed finding summaries and GRADE evidence profiles to clearly describe the benefits and harms (Song et al., 2021). The certainty of the body of evidence was graded as high, moderate, low, or very low. The steering group and GDG thoroughly considered the balance between desirable and undesirable outcomes, certainty of evidence, resource implication, and feasibility. Then, the strength of recommendations was graded as strong for, weak for, weak against, or strong against after a three-round Delphi vote. The evidence and recommendation grading scheme are shown in Supplementary Table S2. Finally, the recommendations draft was sent to the external review group. The steering group revised the recommendations according to suggestions from external reviewers and approved the final guideline.

## Results

Twenty-five clinical questions about off-label drugs used in ophthalmology in China were included. Seventeen drug uses were off-label in the United States and the European Union. A total of 32 systematic reviews, including 24 conducted by the SR group and eight high-relevance published within 2 years, were referred to address these questions. Finally, the guideline developed 32 recommendations, including five strong and 27 weak recommendations for the treatment of ocular fundus, corneal disease, glaucoma, and endophthalmitis. Drug approval status, GRADE evidence profiles, and summaries of findings are shown in the Supplementary Appendix. The characteristics of the systematic reviews included for recommendations formulation are presented in Table 1.

**TABLE 1:** The characteristics of the systematic reviews included for recommendations formulation.

Recommendations for each question		Patients			Interventions
		Diagnose	Age (years)	Female (%)	Usages and dosages
Q1	Recommendation 1	Patients with proliferative, diabetic retinopathy	51.69	41.10%	Intravitreal anti-VEGF drugs, including aflibercept 2 mg, ranibizumab 0.5 mg, or bevacizumab 1.25 mg
Q1	Recommendation 2	Patients with proliferative, diabetic retinopathy	56.60	NA	Intravitreal anti-VEGF drugs before vitrectomy, including conbercept 0.5 mg, ranibizumab 0.5 mg, bevacizumab 1.25 mg
Q2	Recommendation	Patients with retinopathy of prematurity	26.60 weeks	47.10%	Intravitreal anti-VEGF drugs, including ranibizumab 0.1–0.3 mg; bevacizumab 0.625–1.25 mg
Q3	Recommendation	Patients with neovascular glaucoma	54.20	44.32%	Intravitreal anti-VEGF drugs combined with surgery, including aflibercept 2 mg, conbercept 0.5 mg, ranibizumab 0.5 mg, bevacizumab 1.25 mg
Q4	Recommendation	Patients with choroidal neovascularization secondary to pathological myopia	53.38	70.78%	Intravitreal anti-VEGF drugs, including aflibercept 2 mg, ranibizumab 0.5 mg, or bevacizumab 1.25 mg
Q5	Recommendation 1 and 2	Patients with unilateral or bilateral macular oedema secondary to branch retinal vein occlusion or central retinal vein occlusion	64.36	45.34%	Intravitreal anti-VEGF drugs including aflibercept 2 mg, conbercept 0.5 mg, ranibizumab 0.5 mg or 0.3 mg, bevacizumab 1.25 mg
Q6	Recommendation	Patients with Coats’ disease at 2, 3, 4 stages	15.63	22.75%	Intravitreal anti-VEGF drugs or intravitreal anti-VEGF drugs combined with ablation therapy, including conbercept 0.5 mg, ranibizumab 0.5 mg, bevacizumab 1.25 mg
Q7	Recommendation	Patients with uveitis in cataracts, glaucoma, and Behcet’s disease	49.49	51.41%	Anterior chamber injections of TA 0.5, 1.0, 2.0 mg; intravitreal injection of TA 1.0 mg, 4 mg; posterior subtenon injection of TA 40 mg; orbital floor injection of TA 40 mg; suprachoroidal injections of TA 4 mg
Q8	Recommendation	Patients with macular edema due to various causes (including diabetes, uveitis, retinitis pigmentosa, post-surgery, retinal vein occlusion)	59.59	47.63%	Intravitreal,sub-Tenon, suprachoroidalor retrobulbar injection of TA 1, 2 or 4 mg
Q9	Recommendation 1	Patients with VKH disease	44.54	42.28%	Intravenous pulse 40 mg∼1 g methylprednisolone lasts for 3 days sequential oral prednisone/prednisolone 1 mg/kg/d, or 30–100 mg/d
Q9	Recommendation 2	Patients with VKH disease	38.89	36.99%	Intravenous methylprednisolone 200 mg with gradually intravenous dose reduction then gradually oral dose reduction (from 1 mg/kg/d); intraocular injection of TA 4–30 mg
Q10	Recommendation 1	Patients with VKH disease	38.59	44.86%	Prednisone 30 mg/d-1 mg/kg/d plus cyclopsporine 3–5 mg/kg/d
Q10	Recommendation 2	Patients with VKH disease who did not respond well to glucocorticoid	35.67	95.20%	Azathioprine 2–3 mg/kg/d; cyclosprine 3–5 mg/kg/d for at least 1 year
Q11	Recommendation	Patients with Behcet’s disease	30.10	23.17%	Prednisolone 1 mg/kg/d or 12.5 mg–60 mg/d; methylprednisolone 1000 mg for 3 consecutive days followed by oral corticosteroid 0.5 mg/kg
Q12	Recommendation	Patients with optic neuritis	32.18	72.78%	Intravenous methylprednisolone 1 g/day for 3 days followed by oral corticosteroid for 7–11 days
Q13	Recommendation	Patients with acute retinal necrosis	38.04	32.64%	Intravenous acyclovir 10 mg/kg, or 500–750 mg, 3 times/day for 7–14 days; then 3 times/day oral 200 mg, lasts for 4–8 weeks
Q14	Recommendation 1	Glaucoma patients undergoing trabeculectomy	52.60	NA	Intraoperative infiltration, mitomycin 0.2 mg/ml
Q14	Recommendation 2	Glaucoma patients who underwent bleb needling after trabeculectomy failure	64.65	53.09%	Subconjunctival MMC (0.02 ml of 0.2 mg/ml, or 0.1 ml of 0.4 mg/ml) before needling procedure. Subconjunctival MMC (0.1 ml of 0.2 mg/ml) at the end of needling procedure
Q15	Recommendation	Patients with glaucoma	NA	NA	Isosorbide solution, oral doses of 1 g/kg to 2 g/kg
Q16	Recommendation	Patients with malignant (ciliary block) glaucoma	NA	68.93%	1% atropine eye drops
Q17	Recommendation	Patients with corneal transplant	38.89	53.27%	0.03%–0.1% tacrolimus eye drops
Q18	Recommendation 1	Patients with dry eye	48.39	70.87%	0.05% cyclosporine eye drops alone or combined with artificial tears
Q18	Recommendation 2	Patients with dry eye related with Sjögren’s syndrome or graft versus host disease	42.04	70.53%	0.03%–0.1% tacrolimus eye drops alone or combined with artificial tears
Q19	Recommendation	Patients with Mooren ulcers	45.84	39.36%	0.1% tacrolimus eye drops or 1%–2% cyclosporine eye drops
Q20	Recommendation 1	Patients with acute adenoviral keratoconjunctivitis	38.45	55.41%	1% prednisolone acetate four times/day; dexamethasone 0.1% four times/day
Q20	Recommendation 2	Patients with chronic adenoviral keratoconjunctivitis, epidemic keratoconjunctivitis	29.71	48.86%	Fluorometholone eye drops four times/day; dexamethasone 0.05% ointment twice daily
Q21	Recommendation	Patients with infectious endophthalmitis	52.60	28.58%	Intravitreal injection of ceftazidime alone (1–2.25 mg/0.1 ml) or combined with vancomycin (1mg/0.1 ml)
Q22	Recommendation	Patients with infectious endophthalmitis	62.21	67.82%	Intravitreal injection of amikacin (0.4–0.5 mg/0.1 ml) combined with vancomycin (1 mg/0.1 ml)
Q23	Recommendation	Patients with infectious endophthalmitis	67.39	44.49%	Intravitreal injection of vancomycin alone (1 mg/0.1 ml) or combined with amikacin (0.4–0.5 mg/0.1 ml)/ceftazidime (1–2.25 mg/0.1 ml)
Q24	Recommendation	Patients with fungal endophthalmitis	49.19	43.86%	Intravitreal injection of voriconazole (100 µg/0.1 ml)
Q25	Recommendation	Patients with fungal endophthalmitis	44.51	36.70%	Intravitreal injection of amphotericin B (5–10 µg/0.1 ml)

NA, not available; VEGF, vascular endothelial growth factor; TA, triamcinolone acetonide; VKH, Vogt-Koyanagi-Harada.

## Actionable Recommendations

### Q1 on Antagonists of Vascular Endothelial Growth Factor Drugs for Diabetic Retinopathy

Recommendation 1. Anti-VEGF drugs alone or in combination with panretinal laser photocoagulation (PRP), are recommended for the treatment of proliferative diabetic retinopathy (PDR), which can improve best-corrected visual acuity (BCVA), reduce vitrectomy and vitreous bleeding rates (strong recommendation with high certainty evidence).

Evidence. A systematic review and meta-analysis article (*n* = 632, five RCTs, high certainty evidence, Supplementary Table S3) showed that, compared with PRP, anti-VEGF drug had gained better BCVA at 12 months [Mean Difference (MD) = −0.08 logMAR, 95%CI −0.15 to −0.01]. Anti-VEGF monotherapy resulted in a lower incidence of center-involved macular edema at 12 months [Risk Difference (RD) = −0.09, 95%CI −0.19 to 0.00]. The incidence of vitreous hemorrhage was lower [Relative Risk (RR) = 0.72, 95%CI 0.55–0.93], and the vitrectomy rate was lower (RD = −0.09, 95%CI −0.12 to −0.05). (Yates et al., 2021).

Recommendation 2. The application of anti-VEGF in the treatment of PDR before vitrectomy can reduce the incidence of postoperative retinal detachment and the thickness of macular fovea (weak recommendation with moderate certainty evidence).

Evidence. A systematic review and meta-analysis article (*n* = 880 eyes, 11 RCTs, moderate certainty evidence, Supplementary Table S4) revealed that the incidence of postoperative retinal detachment in PDR patients who used anti-VEGF drugs before vitrectomy was significantly lower than that in patients without anti-VEGF drugs treatment (RR = 0.39, 95%CI 0.22–0.71). Patients treated with anti-VEGF gained a lower thickness of macular fovea at 3 and 6 months (MD = −78.49 μm, 95%CI −94.81 to −62.17; MD = -39.62 μm, 95%CI −48.44 to −30.8), and a better BCVA at 6 months after vitrectomy (MD = −0.22 logMAR, 95%CI −0.34 to −0.11). (Hu et al., 2021).

### Q2 on Antagonists of Vascular Endothelial Growth Factor Drugs for Retinopathy of Prematurity

Recommendation. The efficacy and safety of anti-VEGF drugs in treating type I retinopathy of prematurity (ROP) are comparable to those of PRP. Anti-VEGF drugs delay disease recurrence and improve refraction (weak recommendation with low certainty evidence).

Evidence. A systematic review and meta-analysis article (*n* = 3701 eyes, 24 controlled studies involving 8 RCTs, low certainty evidence, Supplementary Table S5) showed that no significant difference was found in the recurrence rate, visual outcome, and ocular adverse event between anti-VEGF drugs and PRP. The time to re-treatment or recurrence after initial treatment was longer in the anti-VEGF drugs group compared to the PRP group (MD = 6.43 weeks, 95%CI 2.36–10.51). Patients using anti-VEGF drugs had a higher proportion of emmetropic eyes (RR = 1.96, 95%CI 1.01–3.70). The meta-analysis results of RCTs were basically consistent with the overall meta-analyses (Popovic et al., 2021).

### Q3 on Antagonists of Vascular Endothelial Growth Factor Drugs for Neovascular Glaucoma

Recommendation. Anti-VEGF drugs in neovascular glaucoma are suggested to improve BCVA and reduce the incidence of surgery-related adverse events (weak recommendation with low certainty evidence).

Evidence. The systematic review conducted by the SR group (*n* = 1189, 14 RCTs, low certainty evidence, Supplementary Table S6) showed that intraocular pressure (IOP) in patients with anti-VEGF drugs combined surgery was significantly decreased in 8 weeks (MD = −6.15 mmHg, 95%CI −9.66 to −2.64), and the BCVA was better in 8 weeks (MD = −0.33logMAR, 95%CI −0.45 to −0.21), compared with surgery alone. Anti-VEGF drugs significantly reduced the risk of surgery-related adverse events (RR = 0.30, 95%CI 0.23–0.40).

### Q4 on Antagonists of Vascular Endothelial Growth Factor Drugs for Choroidal Neovascularization

Recommendation. Anti-VEGF drugs are suggested to use in the treatment of choroidal neovascularization secondary to pathological myopia to improve patients’ visual acuity and reduce central foveal thickness (CFT) (weak recommendation with moderate certainty evidence).

Evidence. The systematic review conducted by the SR group (*n* = 720, 7 RCTs, moderate certainty evidence, Supplementary Table S7) showed that compared with placebo, anti-VEGF drugs significantly improved BCVA (MD = −0.28 logMAR, 95%CI −0.36 to −0.20) and CFT (MD = −66.80 μm, 95%CI −114.87 to −18.73). Compared with photodynamic therapy (PDT), anti-VEGF drugs significantly improved BCVA (MD = −0.14 logMAR, 95%CI −0.17 to −0.10) and CFT (MD = −44.32 μm, 95%CI −59.85 to −28.79). Compared with PDT combination therapy, anti-VEGF monotherapy had similar improvement in BCVA (MD = 0.07 logMAR, 95%CI 0.00 to 0.14) and CFT (MD = 6.40 μm, 95%CI −20.10 to 32.90). However, there was no significant difference in the risk of ocular adverse events and severe adverse events.

### Q5 on Antagonists of Vascular Endothelial Growth Factor Drugs for Macular Edema Secondary to Retinal Vein Occlusion

Recommendation 1. Anti-VEGF drugs are recommended to treat macular edema secondary to retinal vein occlusion to improve visual acuity and reduce macular edema (strong recommendation with high certainty evidence).

Evidence. The systematic review conducted by the SR group (*n* = 310, 16 RCTs, high certainty evidence, Supplementary Table S8) showed that anti-VEGF drugs were significantly associated with better BCVA at 6 and 12 months, compared with placebo (MD = 12.69 letters, 95%CI 8.10–17.28; MD = 7.14 letters, 95%CI 5.43–8.85, respectively). There was no statistical difference in the risk of ocular adverse events between the two groups at 6 months. Anti-VEGF drugs significantly improved visual acuity, compared with PRP (MD = 9.41 letters, 95%CI 6.95–11.86). Anti-VEGF drugs significantly improved visual acuity compared with local ocular injection of glucocorticoids. Further, glucocorticoid was significantly associated with the increased risk of ocular adverse events.

Recommendation 2. Anti-VEGF drugs are equally effective in macular edema secondary to central retinal vein occlusion and branch retinal vein occlusion (strong recommendation with moderate certainty evidence).

Evidence. The systematic review (*n* = 310, 16 RCTs, high certainty evidence, Supplementary Table S8) conducted by the SR group showed that anti-VEGF drugs improved visual acuity and reduced central macular thickness equally in central retinal vein occlusion (CRVO) and branch retinal vein occlusion (BRVO). A systematic review article (*n* = 1631, 8 RCTs, moderate certainty evidence) showed that anti-VEGF drugs were more effective in improving visual acuity and reducing macular edema in patients with branch retinal vein occlusion than placebo, laser photocoagulation, and local ocular injection of glucocorticoids (Shalchi et al., 2020).

### Q6 on Antagonists of Vascular Endothelial Growth Factor Drugs for Coats’ Disease

Recommendation. Anti-VEGF drugs as initial treatment combined with ablation therapy are recommended to improve visual acuity in Coats’ disease, but the monitoring of fibrosis change and other adverse effects should be required (weak recommendation with very low certainty evidence).

Evidence. The systematic review conducted by the SR group (*n* = 378, 24 case series, very low certainty evidence, Supplementary Table S9) found that 16 case series reported the specific visual acuity changes before and after anti-VEGF drugs combined with ablation therapy, involving 194 patients, including 73 (37.63%) with improved visual acuity, 89 (45.87%) with stable visual acuity, 12 (6.19%) with worsening visual acuity, and 20 (10.31%) with unexamined visual acuity. Retinal reattachment after treatment was reported in five studies. Of 57 patients, 42.11% achieved complete retinal reattachment, 38.60% succeeded in partial retinal reattachment, and 19.29% failed in retinal reattachment. Twenty-one studies reported the safety of anti-VEGF drugs combined with ablation therapies in Coats’ disease. Fibrosis change was the most common adverse event (*n* = 35), followed by cataract formation (*n* = 13), and traction retinal detachment (*n* = 12), with no systemic adverse events.

### Q7 on Local Ocular Triamcinolone Acetonide Injection for Uveitis

Recommendation. Local ocular injection of triamcinolone acetonide is suggested to treat uveitis in cataracts, glaucoma, and Behcet’s disease, to improve visual acuity and anterior chamber inflammation. Nevertheless, attention should be paid to monitoring IOP, cataracts, and other adverse events (weak recommendation with low certainty evidence).

Evidence. The systematic review conducted by the SR group (*n* = 839, 2 RCTs, 1 cohort study, 3 case-control studies, 12 case series, low certainty evidence, Supplementary Table S10) found that local ocular injection of triamcinolone acetonide benefitted in improving visual acuity and anterior chamber inflammation. A case-control study reported that the proportion of corrected visual acuity of >0.5 was significantly higher in the triamcinolone acetonide group than that in the control group (29/37 vs. 19/34). Two RCTs showed that the triamcinolone acetonide group significantly improved early postoperative visual acuity compared with the control group. Eight case series reported significant improvement in visual acuity before and after treatment with triamcinolone acetonide. Results of one RCT and one case-control study showed that postoperative anterior chamber inflammation was significantly lower in the triamcinolone acetonide group than that in the control group. Fifteen studies reported different ocular adverse events, including increased IOP, cataracts, and eye pain.

### Q8 on Local Ocular Triamcinolone Acetonide Injection for Macular Edema

Recommendation. Local ocular injection of triamcinolone acetonide is recommended to treat macular edema secondary to diabetes, uveitis, and retinal vein occlusion. Intraocular triamcinolone acetonide can be used as a second-line treatment because its efficacy and safety are inferior to anti-VEGF drugs (strong recommendation with high certainty evidence).

Evidence. The systematic review conducted by the SR group (*n* = 3329, 33 RCTs, high certainty evidence, Supplementary Table S11) showed that no significant difference in BCVA was found between intralocular triamcinolone acetonide injection and placebo. Triamcinolone acetonide significantly reduced central macula thickness compared with placebo at 3 and 6 months (MD = −36.95 µm, 95%CI −48.92 to −24.98; MD = −46.38 µm, 95%CI −69.40 to −23.36, respectively). Compared with anti-VEGF drugs, no statistical difference in BCVA improvement was found at 3, 6, and 12 months. The central macula thickness between the two groups was very similar. There was no significant difference in BCVA improvement or reduction in macular edema between PRP and triamcinolone acetonide. However, triamcinolone acetonide was associated with an increased risk of intraocular hypertension.

### Q9 on Glucocorticoids for Vogt-Koyanagi-Harada Disease

Recommendation 1. It is suggested that intravenous pulse sequential oral glucocorticoid therapy can reduce disease attacks and rapidly improve retinal detachment in VKH disease. Still, attention should be paid to monitoring glucocorticoid-related adverse reactions (weak recommendation with very low certainty evidence).

Evidence. The systematic review conducted by the SR group (*n* = 281 eyes, 1 RCT plus 3 cohort studies, very low certainty evidence, Supplementary Table S12) evaluated the effect of intravenous pulse sequential oral glucocorticoid (IV pulse) *versus* oral glucocorticoids. One retrospective comparative study found that the frequency of uveitis occurrence in the IV pulse group was significantly decreased (number/year 0.23 ± 0.21 vs. 0.40 ± 0.35). Five days after treatment, the maximum retinal detachment height was also considerably lower than that in the oral treatment group. No significant difference was observed in ocular complications such as anterior retinal membrane, neovascularization, and subretinal fibrosis.

Recommendation 2. Systemic glucocorticoid therapy combined with local ocular triamcinolone acetonide injection is suggested to treat VKH disease. Patients may have visual benefits, but attention should be paid to monitoring glucocorticoid-related adverse reactions (weak recommendation with very low certainty evidence).

Evidence. The systematic review conducted by the SR group (*n* = 144 eyes, 3 RCTs, very low certainty evidence, Supplementary Table S13) evaluated the efficacy and safety of systemic glucocorticoid combined with intraocular triamcinolone acetonide, compared with systemic glucocorticoid alone. The results of three RCT studies were inconsistent. One RCT showed a significant improvement in visual acuity within 8 months after the combined therapy. The other two RCTs showed no such benefit at 1 week and 6 months after the combined treatment. Two RCTs showed a significant reduction of neural cortical thickness 8 months after the combined therapy.

### Q10 on Cyclosporine for Vogt-Koyanagi-Harada Disease

Recommendation 1. It is suggested to combine cyclosporine with glucocorticoid for the treatment of VKH disease when the effect of glucocorticoid is not satisfactory (weak recommendation with very low certainty evidence).

Evidence. The systematic review conducted by the SR group (*n* = 177, 3 RCT plus one cohort study, very low certainty evidence, Supplementary Table S14) evaluated the efficacy and safety of cyclosporine therapy plus oral glucocorticoid, compared with glucocorticoid therapy. Two studies (*n* = 60) found that cyclosporine therapy plus oral glucocorticoid gained better visual acuity than glucocorticoid therapy. In both studies (1 RCT and 1 cohort study, *n* = 71), cataracts, glaucoma, and systemic adverse events were reported sporadically in both groups. Two RCTs evaluated the efficacy and safety of cyclosporine plus oral glucocorticoid therapy, compared with intravenous pulse followed by oral glucocorticoid administration. One RCT showed no significant difference in the 12-months recurrence risk between the two groups. In both RCTs, inconsistent visual acuity results were reported, while a significant improvement in fundus lesions was observed in the cyclosporine group. One RCT found that the incidence of cataracts was lower in the cyclosporine group, but there were 3 cases of drug withdrawal due to adverse events, which was higher than in the control group.

Recommendation 2. Cyclosporine is suggested to treat VKH disease in which glucocorticoid therapy does not respond well, and may be as effective and safe as azathioprine (weak recommendation with low certainty evidence).

Evidence. One RCT (Low certainty evidence, Supplementary Table S15) involving 21 patients showed that two of nine in the cyclosporine group were relapsed, and 6 of 12 in the azathioprine group were relapsed (22.2% vs. 50%). The glucocorticoid dosage in the cyclosporine group was significantly lower than that in the azathioprine group. There were no significant differences in visual acuity, anterior chamber inflammation, ocular complications, and non-ocular adverse events between the two groups.

### Q11 on Prednisolone for Behcet’s Disease

Recommendation. Prednisone is suggested to improve ocular inflammation in patients with Behcet’s disease, but attention should be paid to monitoring glucocorticoid-related adverse reactions (weak recommendation with low certainty evidence).

Evidence. The systematic review conducted by the SR group (*n* = 862, 1 RCT plus 4 case series, low certainty evidence, Supplementary Table S16) evaluated the efficacy and safety of prednisolone compared with other treatments. One RCT found a significant visual acuity improvement in the intravenous pulse methylprednisolone group compared with the placebo group. There was no significant difference in safety between the two groups. Almost all patients receiving the intravenous pulse therapy had signs of glucocorticoid excess, such as increased weight or moon faces. In four case series, patients receiving glucocorticoid combined with immunosuppressant responded well, with a significant reduction in ocular inflammatory symptoms and improvement in vision. Most patients experienced recurrence and worsening of visual inflammatory symptoms as the daily steroid dose was gradually tapered.

### Q12 on Systemic Glucocorticoids for Optic Neuritis

Recommendation. Intravenous pulse glucocorticoid therapy is suggested for patients at the acute stage of optic neuritis, but attention should be paid to monitoring glucocorticoid-related adverse reactions. Systemic glucocorticoids are not indicated in convalescent patients (weak recommendation with low certainty evidence).

Evidence. The systematic review article (Gal et al., 2015) (*n* = 693, 6 RCTs, low certainty evidence, Supplementary Table S17) assessed the effects of glucocorticoids on visual recovery in patients with acute optic neuritis. No new studies were included after the updated search by the SR group. No significant difference was found in visual acuity recovery, visual field, and contrast sensitivity in the normal range when comparing oral/intravenous glucocorticoids to placebo. However, the incidence of adverse events, such as depression, acute pancreatitis, and weight gain in the intervention group was significantly higher than placebo. It should be noted that intravenous methylprednisolone significantly improved visual field, contrast sensitivity, and color vision at 6 months compared with placebo in the ONTT study from 1992 to 2006. Nevertheless, no significant difference was observed in visual acuity improvement.

### Q13 on Acyclovir for Acute Retinal Necrosis

Recommendation. Acyclovir combined with laser therapy and surgery in the treatment of acute retinal necrosis can relieve ocular inflammation and reduce the risk of the involvement of the fellow eye in patients (weak recommendation with very low certainty evidence).

Evidence. The SR group conducted the systematic review (*n* = 321, 1 cohort study plus 5 case series, very low certainty evidence, Supplementary Table S18). In all five case series, visual acuity improved after acyclovir treatment combined with laser and surgery. Results in 3 of all case series showed controlled inflammation and improved retinal reattachment. A cohort study showed that the fellow eyes of patients treated with acyclovir were more likely to remain disease-free than those not treated with acyclovir. No adverse events were reported.

### Q14 on Intraoperative and Postoperative Use of Mitomycin C for Glaucoma

Recommendation 1. The use of MMC in trabeculectomy for glaucoma patients can reduce postoperative IOP, form filtering bleb, and decrease the risk of trabeculectomy failure (weak recommendation with moderate certainty evidence).

Evidence. The SR group updated the systematic review (Wilkins et al., 2005) (*n* = 1328, 19 RCTs, moderate certainty evidence, Supplementary Table S19). MMC was significantly better than placebo after trabeculectomy for reducing postoperative IOP (MD = −10.99 mmHg, 95% CI −18.52 to −3.47). MMC was superior to the control group with the formation of filtering bleb (RR = 1.18, 95%CI 1.09 to 1.27). The surgical failure rate was significantly lower with MMC compared with placebo (RR = 0.43, 95%CI 0.28 to 0.66). There was no significant difference observed between MMC and placebo in terms of adverse events of hypotony, anterior chamber bleeding, filter bubble leakage, and endophthalmitis, apart from the shallow anterior chamber.

Recommendation 2. The application of MMC in bleb needling after trabeculectomy failure in glaucoma patients might reduce the postoperative number of anti-glaucoma medications, but lack robust evidence of benefit in lowering IOP and increasing surgical success rate (weak recommendation with low certainty evidence).

Evidence. A systematic review article (Halili et al., 2020) (*n* = 437, 1 RCT plus five observational studies, low certainty evidence, Supplementary Table S20) compared the effects of employing either MMC, 5-Fluorouracil (5-FU), or no anti-metabolite in bleb needling after trabeculectomy. There was no significant difference in IOP (MD = −1.67 mmHg, 95% CI -4.35 to 1.00), surgical success (RR = 1.32; 95%CI 0.72 to 2.39) between MMC group and no anti-metabolite group. However, another systematic review (Chen et al., 2020) (n = 2182, 2 RCTs plus 35 observational studies, moderate certainty evidence, Supplementary Table S21) showed that patients gained better IOP reduction at the last visit (MD = −9.72 mmHg; 95%CI −8.41 to −11.03) and reduced anti-glaucoma medications (MD = 0.80; 95%CI 0.73 to 1.53) after bleb needling surgery with MMC than before.

### Q15 on Isosorbide for Glaucoma

Recommendation. Single-dose oral isosorbide treatment in glaucoma can rapidly reduce IOP (weak recommendation with very low certainty evidence).

Evidence. The systematic reviews conducted by the SR group (*n* = 221, 5 case series, very low certainty evidence, Supplementary Table S22) showed that oral doses of 1 to 2 g/kg isosorbide solution reduced IOP, and the reduction in IOP in glaucoma was more significant than that in normal eyes. A total of 167 patients were included in 4 case series studies, in which 39 adverse events were recorded, with an incidence of 23.35%, mainly headache, mild nausea, dizziness, thirst, moderate pain, diarrhea, and stomach discomfort.

### Q16 on Atropine in the Prophylactic Treatment of Malignant (Ciliary Block) Glaucoma

Recommendation. Topical atropine is suggested to treat malignant (ciliary block) glaucoma to relieve glaucoma-related symptoms (weak recommendation with very low certainty evidence).

Evidence. The SR group conducted the systematic review (*n* = 351, 13 case series, low certainty evidence, Supplementary Table S23). Of 351 eyes in 315 patients included, 58 patients involving 63 eyes with malignant (ciliary block) glaucoma were in remission without surgical treatment after combined treatment with 1% atropine eye drops, with an overall remission rate of 17.95% (5.56%–61.54%). No adverse events were reported.

### Q17 on Tacrolimus Eye Drops for Corneal Transplantation

Recommendation. Tacrolimus eye drops are recommended after corneal transplantation to improve visual acuity and reduce the incidence of graft rejection (strong recommendation with high certainty evidence).

Evidence. The systematic review conducted by the SR group (*n* = 370, 6 RCTs, high certainty evidence, Supplementary Table S24) showed that the incidence of graft rejection after corneal transplantation was significantly decreased when comparing tacrolimus eye drops to other immunosuppressants (RR = 0.59, 95%CI 0.38 to 0.92). Topical tacrolimus could significantly improve postoperative vision (RR = 1.78, 95%CI 1.13 to 2.80). There was no significant difference in safety between groups.

### Q18 on Cyclosporine and Tacrolimus Eye Drops for Severe Dry Eye

Recommendation 1. It is suggested that 0.05% cyclosporine eye drops heal dry eye, improving dry eye symptoms in patients, but the adverse events such as burning pain and stabbing pain should be monitored (weak recommendation with moderate certainty evidence).

Evidence. Studies retrieved did not differentiate between various levels of dry eye severity. The latest systematic review (Tuan et al., 2020) (*n* = 1085, 11 RCTs, moderate certainty evidence, Supplementary Table S25) showed that 0.05% cyclosporine eye drops alone or in combination with artificial tears could improve dry eye symptoms better than artificial tears alone. Still, the incidence of treatment-related adverse events is high, such as burning pain and stabbing pain.

Recommendation 2. Tacrolimus eye drops may be superior to sodium hyaluronate eye drops and comparable to cyclosporine eye drops in treating immune-related dry eye (weak recommendation with low certainty evidence).

Evidence. Studies retrieved did not differentiate between various levels of dry eye severity. The SR group conducted the systematic review (*n* = 130 eyes, 3 RCTs, low certainty evidence, Supplementary Table S26). Two RCTs showed that tacrolimus eye drops combined with sodium hyaluronate in treating Graft-Versus-Host disease-related dry eye were superior to sodium hyaluronate in improving symptoms, prolonging tear film rupture time, and increasing tear secretion. Another RCT found that tacrolimus eye drops were as effective as cyclosporin in treating dry eyes associated with Sjogren’s syndrome.

### Q19 on Tacrolimus and Cyclosporine Eye Drops for Mooren Ulcer

Recommendation. Tacrolimus and cyclosporine eye drops are suggested to treat Mooren ulcers to improve the ulcer healing rate after surgery (weak recommendation with very low certainty evidence).

Evidence. The SR group conducted the systematic review (*n* = 380, 1 cohort study plus 6 case series, very low certainty evidence, Supplementary Table S27). One cohort study showed that 1% cyclosporine eye drops combined with standard therapy improved clinical symptoms and reduced inflammatory response in patients with Mooren ulcers. Six case series demonstrated that tacrolimus or cyclosporine eye drops for Mooren ulcer effectively enhanced ulcer healing rate, from 61% to 100%. One cohort study showed that 1% cyclosporine eye drops reduced the recurrence rate of Mooren ulcer compared with the control group (8% vs. 18%); three case series reported that the patients had no recurrence. One cohort study showed that 1% cyclosporine eye drops reduced the risk of ocular adverse events compared to the control group (9.5% vs. 16.9%); four case series reported ocular adverse events, including increased IOP, glaucoma, cataract, and corneal graft rejection.

### Q20 on Topical Glucocorticoid for Adenoviral Keratoconjunctivitis

Recommendation 1. Topical glucocorticoid may reduce the incidence of subepithelial infiltrates in acute adenovirus keratoconjunctivitis (weak recommendation with low certainty evidence).

Evidence. The systematic review conducted by the SR group (*n* = 145, 2 RCTs plus 1 cohort study, low certainty evidence, Supplementary Table S28) found that the incidence of subepithelial infiltrates in patients with acute adenovirus keratoconjunctivitis was significantly lower compared with artificial tears at 21 and 28 days.

Recommendation 2. Topical glucocorticoid is not superior to immunosuppressants for the treatment of chronic adenovirus keratoconjunctivitis (weak recommendation with moderate certainty evidence).

Evidence. The SR group conducted the systematic review (*n* = 141, 2 RCTs, moderate certainty evidence, Supplementary Table S29). There was no significant difference in subepithelial infiltrate and recurrence of the condition between glucocorticoid and immunosuppressant in patients with chronic adenovirus keratoconjunctivitis. In contrast, the BCVA was decreased, and IOP was increased in patients using glucocorticoids compared with immunosuppressants at 6 months.

### Q21 on Intravitreal Injection of Ceftazidime for Endophthalmitis

Recommendation. Intravitreal injection of ceftazidime alone or combined with vancomycin is suggested as the empirical treatment of infectious endophthalmitis after ocular surgery or trauma. Whereafter, etiological evidence should be acquired timely for targeted therapy (weak recommendation with very low certainty evidence).

Evidence. The SR group conducted the systematic review (*n* = 165, 1 quasi-experimental study plus 4 case series, very low certainty evidence, Supplementary Table S30). Except for one case series representing an unfavorable effect, the other four studies found that intravitreal injection of ceftazidime effectively improved visual acuity for the treatment of endophthalmitis, and the effective rates were 90.5%, 73.5%, 70%, and 83.3%, respectively.

### Q22 on Intravitreal Injection of Amikacin for Endophthalmitis

Recommendation. Intravitreal injection of amikacin combined with vancomycin is suggested as the empirical treatment of posttraumatic or postoperative endophthalmitis. Whereafter, etiological evidence should be acquired timely for targeted therapy (weak recommendation with very low certainty evidence).

Evidence. The systematic review conducted by the SR group (*n* = 115, 5 case series, very low certainty evidence, Supplementary Table S31) showed a significant improvement in visual acuity in all patients with posttraumatic or postoperative endophthalmitis treated with intravitreal amikacin in combination with vancomycin.

### Q23 on Intravitreal Injection of Vancomycin for Endophthalmitis

Recommendation. Intravitreal injection of vancomycin alone or combined with amikacin/ceftazidime is suggested as the empirical treatment of post-surgical or posttraumatic endophthalmitis. Whereafter, etiological evidence should be acquired timely for targeted therapy (weak recommendation with very low certainty evidence).

Evidence. The SR group conducted the systematic review (*n* = 329, 1 cohort study plus 4 case series, very low certainty evidence, Supplementary Table S32). One cohort study showed that the response rate of intravitreal vancomycin injection (visual acuity of ≥0.1, interstitial refractive transparency) was significantly better than gentamicin (50% vs. 16.67%). Four case series found that intravitreal vancomycin plus ceftazidime/amikacin could improve visual acuity.

### Q24 on Intravitreal Injection of Voriconazole for Fungal Endophthalmitis

Recommendation. Intravitreal injection of voriconazole is suggested as the empirical treatment of fungal endophthalmitis. Whereafter, etiological evidence should be acquired timely for targeted therapy (weak recommendation with very low certainty evidence).

Evidence. The systematic review was conducted by the SR group (*n* = 76, 1 RCT plus 6 case series, very low certainty evidence, Supplementary Table S33). One RCT revealed that after vitreous injection of voriconazole 1 day after vitrectomy, corneal opacity, aqueous humor flash, and vitreous opacity of patients were significantly improved better than those of the control group. Six case series demonstrated that intravitreal injection of voriconazole improved visual acuity.

### Q25 on Intravitreal Injection of Amphotericin B for Fungal Endophthalmitis

Recommendation. Intravitreal injection of amphotericin B is suggested as the empirical treatment of fungal endophthalmitis. Whereafter, etiological evidence should be acquired timely for targeted therapy (weak recommendation with very low certainty evidence).

Evidence. The systematic review was conducted by the SR group (*n* = 297, 1 RCT plus 12 case controls, very low certainty evidence, Supplementary Table S34). In 1 RCT, the corneal turbidity, aqueous humor flash, and vitreous turbidity of patients were improved significantly better than those of the control group after intravitreal injection of amphotericin B. Improvement in visual acuity was reported in 75.0%, 43.4%, 100%, 37.0%, 70%, and 88.2% of patients in 6 case series, respectively.

## Discussion

Chinese evidence-based guideline for off-label drug use in ophthalmology was developed using the rigorous and multidisciplinary method of WHO guideline development. The strategy generated five strong recommendations and 27 weak recommendations to address 25 clinical questions, covering the most common and rare diseases in ophthalmology, such as ocular fundus, corneal disease, glaucoma, dry eye, Coat’s disease, Behcet’s disease, and endophthalmitis. Medications that were recommended to treat the above-mentioned diseases, included anti-VEGF agents, immunosuppressive drugs, glucocorticoids, and antimicrobial agents. The guideline recommendations were consistent with the international guidelines and consensuses developed in America, Europe, and Japan, further regulating rational and optimized uses of the ophthalmic drug.

Since the outbreak of the COVID-19 pandemic, there have been multiple attempts to use medications already approved or developed for indications, which are repurposed for the treatment of COVID-19 based on the assumptions of safety and efficacy with short approval time (Guy et al., 2020; Shaffer, 2020). According to the literature review, off-label drugs used in ophthalmology are frequent, and they mainly include anti-vascular endothelial growth factors used in neovascularization disease (Dascalu et al., 2016; Bro et al., 2020; Chakraborty et al., 2021; Panigrahi, 2021). Nevertheless, in general, as well as in the treatment of ocular surface diseases in particular, we benefit significantly from the off-label use of drugs that are first developed for other indications (Novack, 2021). These medications include glucocorticoids, anti-infectious agents, and immunosuppressive drugs, indicating that conditions in ophthalmology are essentially similar to the conditions in systemic medicine. Therefore, off-label drugs use in ophthalmology is widespread in clinical practice, and even it is used as a conventional treatment regardless of the quantity and quality of evidence. Our guideline provided recommendations for those clinical questions applying the current best available evidence and expert’s clinical opinions.

In regulating the off-label use of approved drugs, the Food and Drug Administration (FDA) controls the commercial availability of novel therapeutics but does not restrict physician prescriptions. Consequently, the physician may legally prescribe approved agents for unapproved indications (i.e., off-label) based on their best clinical judgement (Mello et al., 2009; Kesselheim et al., 2019). China passed the Physician Law in August 2021 to regulate off-label prescriptions for the first time. The new law, set to take effect on 1 March 2022, will grant physicians the right to use off-label drugs, which will allow physicians to use off-label drugs with the instruction of current guidelines when there are no effective or better accessible treatments for specific conditions.

To our knowledge, this was the first evidence-based guideline for off-label drugs used in ophthalmology in China. The guideline has several strengths. Firstly, we followed the internationally recognized guideline development methodology, ensuring the guideline’s validity, reliability, and reproducibility. For example, we registered the guideline online, performed a systematic review for each clinical question, used the GRADE approach and Delphi vote to generate recommendations, conducted an external peer review on the recommendations, and reported the guideline according to the RIGHT statement. Secondly, we emphasized the management of conflicts of interest and involvement with frontline medical workers in the guideline development, which enhanced the transparency and applicability of our guideline. Thirdly, our guideline provided a practical recommendation regarding the off-label drugs used for many refractory eye diseases, including anti-VEGF agents, immunosuppressive drugs, glucocorticoids, and antimicrobial agents, to promote the rational use of drugs.

The guideline also has limitations. Firstly, off-label drug usages in the guideline are mainly for China, which might have been approved in other countries. For example, only 17 usages in the guideline are off-label in the United States and the European Union. However, this does not affect applying the guideline as a reference for medical professionals in these countries. Secondly, some recommendations for off-label drug use are only supported with low-quality evidence and formulated more based on expert opinions, which may bring some bias. However, we had adopted the Delphi method of voting to minimize the bias.

In conclusion, we developed an evidence-based guideline concerning off-label drug use in ophthalmology using a rigorous and multidisciplinary approach. The guideline provides more comprehensive recommendations for informing clinical care, which is of greater applicability. However, considering the lack of robust evidence for some off-label usages, more high-quality extensive observational studies and RCTs are still urgently needed.
