# Supplementary material for: Recommendations for Off-Label Drug Use in Ophthalmology in China: A Clinical Practice Guideline
Source: Front Pharmacol. 2022 May 24;13:919688. doi: 10.3389/fphar.2022.919688 (PMC9171102; doi:10.3389/fphar.2022.919688)
Supplement: Supplementary file 1 [file DataSheet1.docx]

**Supplementary Appendix**

**Contents**

[eTable 1. The Detail List of Guideline Working Members 4](#_Toc99544879)

[eTable 2. GRADE System Used for Recommendations of the Clinical Practice Guidelines 8](#_Toc99544880)

[Q1 on anti-VEGF drugs for diabetic retinopathy 9](#_Toc99544881)

[eTable 3. Summary of Findings and Certainty of Evidence Using GRADE Approach^1^ 9](#_Toc99544882)

[eTable 4. Summary of Findings and Certainty of Evidence Using GRADE Approach^2^ 10](#_Toc99544883)

[Q2 on anti-VEGF drugs for retinopathy of prematurity 11](#_Toc99544884)

[eTable 5. Summary of Findings and Certainty of Evidence Using GRADE Approach based on included RCTs^1^ 11](#_Toc99544885)

[Q3 on anti-VEGF drugs for neovascular glaucoma 12](#_Toc99544886)

[eTable 6. Summary of Findings and Certainty of Evidence Using GRADE Approach 12](#_Toc99544887)

[Q4 on anti-VEGF drugs for choroidal neovascularization 14](#_Toc99544888)

[eTable 7. Summary of Findings and Certainty of Evidence Using GRADE Approach 14](#_Toc99544889)

[Q5 on anti-VEGF drugs for macular edema secondary to retinal vein occlusion 16](#_Toc99544890)

[eTable 8. Summary of Findings and Certainty of Evidence Using GRADE Approach 16](#_Toc99544891)

[Q6 on anti-VEGF drugs for Coats' disease 19](#_Toc99544892)

[eTable 9. Summary of Findings and Certainty of Evidence Using GRADE-CERQual Approach 19](#_Toc99544893)

[Q7 on local ocular triamcinolone acetonide injection for uveitis 22](#_Toc99544894)

[eTable 10. Summary of Findings and Certainty of Evidence Using GRADE-CERQual Approach approach 22](#_Toc99544895)

[Q8 on local ocular triamcinolone acetonide injection for macular edema 24](#_Toc99544896)

[eTable 11. Summary of Findings and Certainty of Evidence Using GRADE Approach 24](#_Toc99544897)

[Q9 on glucocorticoids for Vogt-Koyanagi-Harada (VKH) disease 28](#_Toc99544898)

[eTable 12. Summary of Findings and Certainty of Evidence Using GRADE-CERQual Approach 28](#_Toc99544899)

[eTable 13. Summary of Findings and Certainty of Evidence Using GRADE-CERQual Approach 29](#_Toc99544900)

[Q10 on cyclosporine for VKH disease 30](#_Toc99544901)

[eTable 14. Summary of Findings and Certainty of Evidence Using GRADE-CERQual Approach 30](#_Toc99544902)

[eTable 15. Summary of Findings and Certainty of Evidence Using GRADE-CERQual Approach 31](#_Toc99544903)

[Q11 on prednisolone for Behcet's disease 33](#_Toc99544904)

[eTable 16. Summary of Findings and Certainty of Evidence Using GRADE-CERQual Approach 33](#_Toc99544905)

[Q12 on systemic glucocorticoids for optic neuritis 34](#_Toc99544906)

[eTable 17. Summary of Findings and Certainty of Evidence Using GRADE Approach^1^ 34](#_Toc99544907)

[Q13 on acyclovir for acute retinal necrosis 36](#_Toc99544908)

[eTable 18. Summary of Findings and Certainty of Evidence Using GRADE-CERQual Approach 36](#_Toc99544909)

[Q14 on intraoperative and postoperative use of mitomycin C (MMC) for glaucoma 37](#_Toc99544910)

[eTable 19. Summary of Findings and Certainty of Evidence Using GRADE Approach 37](#_Toc99544911)

[eTable 20. Summary of Findings and Certainty of Evidence Using GRADE Approach^21^ 38](#_Toc99544912)

[eTable 21. Summary of Findings and Certainty of Evidence Using GRADE-CERQual Approach^22^ 38](#_Toc99544913)

[Q15 on isosorbide for glaucoma 41](#_Toc99544914)

[eTable 22. Summary of Findings and Certainty of Evidence Using GRADE-CERQual Approach 41](#_Toc99544915)

[Q16 on atropine in the prophylactic treatment of malignant (ciliary block) glaucoma 42](#_Toc99544916)

[eTable 23. Summary of Findings and Certainty of Evidence Using GRADE-CERQual Approach 42](#_Toc99544917)

[Q17 on tacrolimus eye drops for corneal transplantation 43](#_Toc99544918)

[eTable 24. Summary of Findings and Certainty of Evidence Using GRADE Approach 43](#_Toc99544919)

[Q18 on cyclosporine and tacrolimus eye drops for severe dry eye 45](#_Toc99544920)

[eTable 25. Summary of Findings and Certainty of Evidence Using GRADE Approach^1^ 45](#_Toc99544921)

[eTable 26. Summary of Findings and Certainty of Evidence Using GRADE-CERQual Approach 45](#_Toc99544922)

[Q19 on tacrolimus and cyclosporine eye drops for Mooren ulcer 47](#_Toc99544923)

[eTable 27. Summary of Findings and Certainty of Evidence Using GRADE-CERQual Approach 47](#_Toc99544924)

[Q20 on topical glucocorticoid for adenoviral keratoconjunctivitis 48](#_Toc99544925)

[eTable 28. Summary of Findings and Certainty of Evidence Using GRADE Approach 48](#_Toc99544926)

[eTable 29. Summary of Findings and Certainty of Evidence Using GRADE Approach 48](#_Toc99544927)

[Q21 on intravitreal injection of ceftazidime for endophthalmitis 50](#_Toc99544928)

[eTable 30. Summary of Findings and Certainty of Evidence Using GRADE-CERQual Approach 50](#_Toc99544929)

[Q22 on intravitreal injection of amikacin for endophthalmitis 51](#_Toc99544930)

[eTable 31. Summary of Findings and Certainty of Evidence Using GRADE-CERQual Approach 51](#_Toc99544931)

[Q23 on intravitreal injection of vancomycin for endophthalmitis 52](#_Toc99544932)

[eTable 32. Summary of Findings and Certainty of Evidence Using GRADE-CERQual Approach 52](#_Toc99544933)

[Q24 on intravitreal injection of voriconazole for fungal endophthalmitis 53](#_Toc99544934)

[eTable 33. Summary of Findings and Certainty of Evidence Using GRADE-CERQual Approach 53](#_Toc99544935)

[Q25 on intravitreal injection of amphotericin B for fungal endophthalmitis 54](#_Toc99544936)

[eTable 34. Summary of Findings and Certainty of Evidence Using GRADE-CERQual Approach 54](#_Toc99544937)

### Table S1. The Detail List of Guideline Working Members

| **Name** | **Title** | **Affiliation** | **Major** |
| --- | --- | --- | --- |
| **the Steering Group** | | | |
| Ningli Wang | Chief Physician | Beijing Tongren Hospital, Capital Medical University | Ophthalmology |
| Wenbin Wei | Chief Physician | Beijing Tongren Hospital, Capital Medical University | Ophthalmology |
| Hai Lu | Chief Physician | Beijing Tongren Hospital, Capital Medical University | Ophthalmology |
| Suodi Zhai | Chief Pharmacist | Peking University Third Hospital | Clinical Pharmacy, Guideline Development Methodologist |
| Chao Zhang | Chief Pharmacist | Beijing Tongren Hospital, Capital Medical University | Clinical Pharmacy |
| Yu Zhang | Chief Pharmacist | Union Hospital, Tongji Medical College, Huazhong University of Science and Technology | Clinical Pharmacy |
| **the Guideline Development Group** | | | |
| Yong Tao | Chief Physician | Beijing Chaoyang Hospital, Capital Medical University | Ophthalmology |
| Zhiqiang Pan | Chief Physician | Beijing Tongren Hospital, Capital Medical University | Ophthalmology |
| Tao Wang | Chief Physician | Beijing Tongren Hospital, Capital Medical University | Ophthalmology |
| Hua Yan | Chief Physician | Tianjin Medical University | Ophthalmology |
| Jianjiang Xu | Chief Physician | Eye and ENT Hospital of Fudan University | Ophthalmology |
| Yalin Dong | Chief Pharmacist | the First Affiliated Hospital of Xi 'an Jiaotong University | Clinical Pharmacy |
| Ruichen Guo | Chief Pharmacist | Qilu Hospital of Shandong University | Clinical Pharmacy |
| Jianguo Zhu | Chief Pharmacist | the First Affiliated Hospital of Soochow University | Clinical Pharmacy |
| Xin Hu | Chief Pharmacist | Beijing Hospital | Clinical Pharmacy |
| Zhigang Zhao | Chief Pharmacist | Beijing Tiantan Hospital, Capital Medical University | Clinical Pharmacy |
| Dan Mei | Chief Pharmacist | Peking Union Medical College Hospital | Clinical Pharmacy |
| Xiujuan Fu | Chief Pharmacist | The Second Hospital of Jilin University | Clinical Pharmacy |
| Yinfei Yu | Chief Pharmacist | The Eye Hospital of Wenzhou Medical University | Clinical Pharmacy |
| Ruizhong Gong | Chief Pharmacist | Shanxi Eye Hospital | Clinical Pharmacy |
| Junjie Zhang | Chief Pharmacist | People's Hospital of Henan University | Clinical Pharmacy |
| Chen Shi | Chief Pharmacist | Union Hospital, Tongji Medical College, Huazhong University of Science and Technology | Clinical Pharmacy |
| Jiuhong Wu | Professor | Special Medical Center of PLA Strategic Support Forces | Pharmacoeconomics |
| Siyan Zhan | Professor | the School of Public Health, Peking University | Pharmacoepidemiology |
| Luwen Shi | Professor | School of Pharmaceutical Sciences, Peking University | Pharmaceutical Administration Policy |
| Yaolong Chen | Professor | Evidence-Based Medicine Center, Lanzhou University | Guideline Development Methodologist |
| Kairong Wang | Lawer | Beijing Liuli Law Firm | Health Law |
| **the External Review Group** | | | |
| Qiang Zhou | Associate Chief Physician | Beijing Chaoyang Hospital, Capital Medical University | Ophthalmology |
| Jing Feng | Associate Chief Physician | Beijing Chaoyang Hospital, Capital Medical University | Ophthalmology |
| Li Chen | Associate Chief Physician | Beijing Chaoyang Hospital, Capital Medical University | Ophthalmology |
| Xiaofeng Hu | Associate Chief Physician | Beijing Chaoyang Hospital, Capital Medical University | Ophthalmology |
| Hao Kang | Associate Chief Physician | Beijing Chaoyang Hospital, Capital Medical University | Ophthalmology |
| Yujing Yang | Doctor-in-Charge | Eye and ENT Hospital of Fudan University | Ophthalmology |
| Qihua Le | Associate Professor | Eye and ENT Hospital of Fudan University | Ophthalmology |
| Xinhan Cui | Doctor-in-Charge | Eye and ENT Hospital of Fudan University | Ophthalmology |
| Hui Zhao | Associate Chief Physician | Shanghai First People's Hospital | Ophthalmology |
| Jun Zou | Chief Physician | Shanghai Tenth People's Hospital | Ophthalmology |
| Zhiwen Fu | Clinical Pharmacist | Union Hospital, Tongji Medical College, Huazhong University of Science and Technology | Clinical Pharmacy |
| Xinghua Wang | Doctor-in-Charge | Union Hospital, Tongji Medical College, Huazhong University of Science and Technology | Ophthalmology |
| Cong Wang | Pharmacist-in-Charge | Union Hospital, Tongji Medical College, Huazhong University of Science and Technology | Clinical Pharmacy |
| Huiping Hu | Pharmacist-in-Charge | Union Hospital, Tongji Medical College, Huazhong University of Science and Technology | Clinical Pharmacy |
| Xuegu Xu | Pharmacist-in-Charge | The Eye Hospital of Wenzhou Medical University | Clinical Pharmacy |
| Pengyuan Du | Clinical Pharmacist | The Eye Hospital of Wenzhou Medical University | Clinical Pharmacy |
| Yuqin Wang | Chief Physician | The Eye Hospital of Wenzhou Medical University | Ophthalmology |
| Peizhen Lin | Pharmacist-in-Charge | The Eye Hospital of Wenzhou Medical University | Clinical Pharmacy |
| Zuhua Sun | Doctor-in-Charge | The Eye Hospital of Wenzhou Medical University | Ophthalmology |
| Junfeng Wang | Chief Pharmacist | Shanxi Eye Hospital | Clinical Pharmacy |
| Chen Wang | Associate Chief Pharmacist | Shanxi Eye Hospital | Clinical Pharmacy |
| Dongping Zheng | Chief Physician | Shanxi Eye Hospital | Ophthalmology |
| Li Wang | Associate Chief Pharmacist | Shanxi Eye Hospital | Clinical Pharmacy |
| Xiaofen Zheng | Chief Physician | Shanxi Eye Hospital | Ophthalmology |
| Jingjing Yang | Pharmacist-in-Charge | People's Hospital of Henan University | Ophthalmic Pharmacotherapy |
| Ruixing Liu | Doctor-in-Charge | People's Hospital of Henan University | Ophthalmology |
| Tianyang Zhou | Associate Chief Pharmacist | People's Hospital of Henan University | Clinical Pharmacy |
| Zheng Yuan | Pharmacist-in-Charge | People's Hospital of Henan University | Ophthalmology |
| Zhanrong Li | Associate Chief Physician | People's Hospital of Henan University | Ophthalmology |
| **the Systematic Review Team** | | | |
| Guangyao Li | Clinical Pharmacist | Beijing Tongren Hospital, Capital Medical University | Clinical Pharmacy |
| Xin Wang | Associate Chief Pharmacist | Beijing Tongren Hospital, Capital Medical University | Clinical Pharmacy |
| Zhihui Song | Associate Chief Pharmacist | Beijing Tongren Hospital, Capital Medical University | Clinical Pharmacy |
| Shanshan Xu | Pharmacist-in-Charge | Beijing Tongren Hospital, Capital Medical University | Clinical Pharmacy |
| Ying Bai | Pharmacist-in-Charge | Beijing Tongren Hospital, Capital Medical University | Clinical Pharmacy |
| Zichao Ji | Pharmacist-in-Charge | Beijing Tongren Hospital, Capital Medical University | Clinical Pharmacy |
| Pengpeng Liu | Pharmacist-in-Charge | Beijing Tongren Hospital, Capital Medical University | Clinical Pharmacy |
| Shuo Feng | Clinical Pharmacist | Beijing Tongren Hospital, Capital Medical University | Clinical Pharmacy |
| Yue Xie | Clinical Pharmacist | Beijing Tongren Hospital, Capital Medical University | Clinical Pharmacy |
| Lu Sun | Clinical Pharmacist | Beijing Tongren Hospital, Capital Medical University | Clinical Pharmacy |
| Liming Dong | Clinical Pharmacist | Beijing Tongren Hospital, Capital Medical University | Clinical Pharmacy |
| Jie Bai | Clinical Pharmacist | Beijing Tongren Hospital, Capital Medical University | Clinical Pharmacy |
| Xiao Cheng | Clinical Pharmacist | Beijing Tongren Hospital, Capital Medical University | Clinical Pharmacy |
| Yiman Li | Clinical Pharmacist | Beijing Tongren Hospital, Capital Medical University | Clinical Pharmacy |
| Ente Wang | Clinical Pharmacist | Beijing Tongren Hospital, Capital Medical University | Clinical Pharmacy |
| Yifei Xue | Master Candidate | Beijing Tongren Hospital, Capital Medical University | Clinical Pharmacy |

### Table S2. GRADE System Used for Recommendations of the Clinical Practice Guideline

| **Certainty of evidence** | |
| --- | --- |
| **Grades** | **Description** |
| High | We are very confident that the true effect lies close to that of the estimate of the effect. |
| Moderate | We are moderately confident in the effect estimate: The true effect is likely to be close to the estimate of the effect, but there is a possibility that it is substantially different. |
| Low | Our confidence in the effect estimate is limited: The true effect may be substantially different from the estimate of the effect. |
| Very low | We have very little confidence in the effect estimate: The true effect is likely to be substantially different from the estimate of effect. |
| **Strength of recommendation** | |
| **Grades** | **Description** |
| Strong for an intervention | Most individuals in this situation would want the recommended course of action; most clinicians would recommend it; it can be adopted as policy in most situations. |
| Weak for an intervention | The majority of individuals in this situation would want the suggested course of action, but many would not; clinicians would recognize that different choices will be appropriate for different patients; policy-making will require substantial debate and involvement of many stakeholders. |
| Weak against an intervention | The majority of individuals in this situation would be against the suggested course of action, but many would not; clinicians would recognize that different choices will be appropriate for different patients; policy-making will require substantial debate and involvement of many stakeholders. |
| Strong against an intervention | Most individuals in this situation would be against the recommended course of action; most clinicians would not recommend it; it can be adopted as policy in most situations. |

## Q1 on anti-VEGF drugs for diabetic retinopathy

**Recommendation 1**

**PICO framework**

Patient: patients with proliferative, diabetic retinopathy

Intervention: anti-VEGF drugs, including aflibercept, conbercept, ranibizumab, bevacizumab, and brolucizumab

Comparison: panretinal laser photocoagulation

Outcome: all outcomes of interest were listed in the eTable below.

### Table S3. Summary of Findings and Certainty of Evidence Using GRADE Approach^1^

| **Quality assessment** | | | | | | | | **Summary of findings** | | | |
| --- | --- | --- | --- | --- | --- | --- | --- | --- | --- | --- | --- |
|  |  |  |  |  |  |  |  | **Number of patients** | | **Effect size (95%CI)** | **Certainty of evidence** |
| **Outcome** | **Number of included studies** | **Study design** | **Risk of bias ^a^** | **Inconsistency ^b^** | **Indirectness ^c^** | **Imprecision ^d^** | **Publication bias ^e^** | **Intervention group** | **Control group** |  |  |
| BCVA at 12 months | 4 | RCT | General | General | None | General | None | 259 | 273 | MD = -0.08 logMAR (-0.15, -0.01) | High |
| Center-involved macular edema at 12 months | 2 | RCT | General | General | None | General | None | 303 | 312 | RD = -0.09 (-0.19, 0.00) | High |
| Vitreous hemorrhage | 5 | RCT | General | None | None | None | None | 10/332 | 41/345 | RR = 0.72 (0.55, 0.93) | High |
| Vitrectomy | 4 | RCT | General | None | None | General | None | 62/323 | 93/332 | RD = -0.09 (-0.12, -0.05) | High |

Abbreviations: BCVA=best corrected visual acuity; RCT=randomized controlled trial; MD=mean difference; RD=risk difference.

Explanation:

a. Risk of bias: the included study did not report hidden allocation, random method and blind method, and was rated as having Serious risk of bias, which was downgraded one level;

b. Inconsistency: if I^2^ is greater than 75% and there is serious inconsistency, it shall be graded as serious, and downgraded one level;

c. Indirectness: if included studies are poor matching with clinical questions, it shall be graded as serious, and downgraded one level;

d. Imprecision: if the number of events included in the study is small, and the sample size is lower than the minimum sample size, it shall be graded as serious, and downgraded one level;

e. Publication bias: insufficient number of included literatures /egger test results P < 0.05, indicates a large risk of publication bias, which shall be graded as serious, and downgraded one level.

The overall quality of quantitative evidence for each outcome of the other clinical questions was graded considering above factors using GRADE approach.

**Recommendation 2**

**PICO framework**

Patient: patients with proliferative, diabetic retinopathy

Intervention: anti-VEGF drugs before vitrectomy, including aflibercept, conbercept, ranibizumab, bevacizumab, brolucizumab

Comparison: vitrectomy alone

Outcome: all outcomes of interest were listed in the eTable below.

### Table S4. Summary of Findings and Certainty of Evidence Using GRADE Approach^2^

| **Quality assessment** | | | | | | | | **Summary of findings** | | | |
| --- | --- | --- | --- | --- | --- | --- | --- | --- | --- | --- | --- |
|  |  |  |  |  |  |  |  | **Number of patients with eye** | | **Effect size (95%CI)** | **Certainty of evidence** |
| **Outcomes** | **Number of included studies** | **Study design** | **Risk of bias** | **Inconsistency** | **Indirectness** | **Imprecision** | **Publication bias** | **Intervention group** | **Control group** |  |  |
| Retinal detachment | 8 | RCT | General | None | None | None | None | 13/349 | 34/327 | RR = 0.39 (0.22, 0.71) | High |
| Thickness of macular fovea at 3 months | 2 | RCT | General | None | None | None | Serious | 84 | 84 | MD = -78.49 μm (-94.81, -62.17) | Moderate |
| Thickness of macular fovea at 6 months | 2 | RCT | General | None | None | Serious | Serious | 107 | 107 | MD = -39.62 μm (-48.44, -30.80) | Low |
| BCVA at 6 months | 6 | RCT | General | General | None | Serious | None | 270 | 276 | MD = -0.22 logMAR (-0.34, -0.11) | Moderate |

Abbreviations: BCVA=best corrected visual acuity; RCT=randomized controlled trial; MD=mean difference; RR=relative risk.

**Drug approval status in the United States, the European Union, and China**

**In the United States,** ranibizumab was approved for the treatment of diabetic retinopathy in April 2017. Aflibercept was approved for the treatment of diabetic retinopathy in May 2019, and for the treatment of diabetic retinopathy in patients with diabetic macular edema in March 2015.

**In the European Union,** ranibizumab was approved for the treatment of proliferative diabetic retinopathy in October 2019.

**In China,** ranibizumab was approved for the treatment of moderate to severe non-proliferative diabetic retinopathy and proliferative diabetic retinopathy in August 2021.

**Reference:**

1. Yates WB, Mammo Z, Simunovic MP. Intravitreal anti-vascular endothelial growth factor versus panretinal LASER photocoagulation for proliferative diabetic retinopathy: a systematic review and meta-analysis. Can J Ophthalmol. 2021;56(6):355-363.
2. Hu WQ，Ji XP， Zhou XB，et al. Meta-analysis of long-term efficacy of vitrectomy combined with anti - VEGF in the treatment of proliferative diabetic retinopathy. Guoji Yanke Zazhi(Int Eye Sci) 2021;21(6):1040-1046.

## Q2 on anti-VEGF drugs for retinopathy of prematurity

**Recommendation**

**PICO framework**

Patients: patients with retinopathy of prematurity

Intervention: anti-VEGF drugs, including aflibercept, conbercept, ranibizumab, bevacizumab, brolucizumab

Comparison: panretinal laser photocoagulation

Outcome: all outcomes of interest were listed in the eTable below.

### Table S5. Summary of Findings and Certainty of Evidence Using GRADE Approach based on included RCTs^1^

| **Quality assessment** | | | | | | | | **Summary of findings** | | | |
| --- | --- | --- | --- | --- | --- | --- | --- | --- | --- | --- | --- |
|  |  |  |  |  |  |  |  | **Number of patients** | | **Effect size (95%CI)** | **Certainty of evidence** |
| **Outcomes** | **Number of included studies** | **Study design** | **Risk of bias** | **Inconsistency** | **Indirectness** | **Imprecision** | **Publication bias** | **Intervention group** | **Control group** |  |  |
| Recurrence rate | 6 | RCT | General | Serious | None | Serious | General | 621/736 | 412/496 | RR = 1.05 (0.91, 1.21) | Low |
| Number of additional treated eyes required | 5 | RCT | General | Serious | None | None | General | 143/596 | 58/350 | RR = 2.81 (0.78, 10.09) | Moderate |
| Time to re-treatment or recurrence | 3 | RCT | General | Serious | None | None | Serious | 268 | 276 | MD = 6.83 w (1.64, 11.13) | Low |
| Vitrectomy or scleral buckling | 3 | RCT | General | None | None | None | Serious | 3/380 | 15/296 | RR = 0.25 (0.05, 1.22) | Moderate |
| Retinal detachment | 3 | RCT | General | None | None | Serious | Serious | 5/301 | 6/455 | RR = 1.28 (0.41, 4.02) | Low |
| Vitreous hemorrhage | 2 | RCT | General | General | None | Serious | Serious | 8/348 | 2/188 | RR = 1.30 (0.03, 49.78) | Low |
| Retinal hemorrhage | 2 | RCT | General | None | None | Serious | Serious | 32/348 | 16/188 | RR = 0.90 (0.34, 2.38) | Low |
| Cataract | 3 | RCT | General | None | None | Serious | Serious | 2/592 | 3/362 | RR = 0.63 (0.10, 3.78) | Low |

Abbreviations: BCVA=best corrected visual acuity; RCT=randomized controlled trial; MD=mean difference; RR=relative risk.

**Drug approval status in the United States, the European Union, and China**

**In the United States,** none.

**In the European Union,** ranibizumab was approved for the treatment of zone I (stage 1+, 2+, 3 or 3+), Zone II (Stage 3+) ROP or acute posterior ROP in September 2019.

**In China,** ranibizumab was approved for the treatment of zone I (stage 1+, 2+, 3 or 3+), Zone II (Stage 3+) ROP or acute posterior ROP in August 2021.

**Reference:**

1. Popovic MM, Nichani P, Muni RH, Mireskandari K, Tehrani NN, Kertes PJ. Intravitreal anti-vascular endothelial growth factor injection versus laser photocoagulation for retinopathy of prematurity: A meta-analysis of 3,701 eyes. Surv Ophthalmol. 2021;66(4):572-584.

## Q3 on anti-VEGF drugs for neovascular glaucoma

**Recommendation**

**PICO framework**

Patients: patients with neovascular glaucoma

Interventions: anti-VEGF drugs combined surgery, including aflibercept, conbercept, ranibizumab, bevacizumab, brolucizumab

Comparison: surgery alone

Outcome: all outcomes of interest were listed in the eTable below

### Table S6. Summary of Findings and Certainty of Evidence Using GRADE Approach

| **Quality assessment** | | | | | | | | **Summary of findings** | | | |
| --- | --- | --- | --- | --- | --- | --- | --- | --- | --- | --- | --- |
|  |  |  |  |  |  |  |  | **Number of patients** | | **Effect size (95%CI)** | **Certainty of evidence** |
| **Outcomes** | **Number of included studies** | **Study design** | **Risk of bias** | **Inconsistency** | **Indirectness** | **Imprecision** | **Publication bias** | **Anti-VEGF drugs group** | **No anti-VEGF drugs group** |  |  |
| IOP in 8 weeks^1-11^ | 11 | RCT | General | General | None | General | Serious | 473 (515 eyes) | 473 (506 eyes) | MD = -6.15mmHg (-9.66, -2.64) | Moderate |
| IOP remission rate in 8 weeks^4,7,8^ | 3 | RCT | General | None | None | General | Serious | 182 (219 eyes) | 182 (211 eyes) | RR = 1.26 (1.16, 1.36) | Moderate |
| IOP remission rate at 1 year^1,2^ | 2 | RCT | General | None | None | Serious | Serious | 40 | 40 | RR = 1.27 (0.90, 1.80) | Low |
| BCVA in 8 weeks^5,6^ | 2 | RCT | General | None | None | Serious | Serious | 40 | 40 | MD = -0.33 logMAR (-0.45, -0.21) | Low |
| Hyphema^1-4,6,7,9,11,12^ | 9 | RCT | General | Serious | None | Serious | General | 352 | 352 | RR = 0.21 (0.13, 0.33) | Low |
| Corneal edema^1,3,4,7,11^ | 5 | RCT | General | None | None | Serious | Serious | 232 | 232 | RR = 0.36 (0.17, 0.75) | Low |
| Eye pain^3,4,13^ | 3 | RCT | General | None | None | Serious | Serious | 165 | 165 | RR = 0.24 (0.09, 0.67) | Low |
| Anterior chamber exudation^3,4,7^ | 3 | RCT | General | None | None | Serious | Serious | 172 | 172 | RR = 0.39 (0.22, 0.70) | Low |
| Choroidal effusion^1,2^ | 2 | RCT | General | None | None | Serious | Serious | 40 | 40 | RR = 0.67 (0.21, 2.15) | Low |
| Conjunctival edema^7,12^ | 2 | RCT | General | General | None | Serious | Serious | 84 | 84 | RR = 0.84 (0.23, 3.00) | Low |
| Encapsulated filtering blebs^6,9^ | 2 | RCT | General | None | None | Serious | Serious | 50 | 50 | RR = 0.33 (0.05, 2.03) | Low |
| Detachment of choroid^6,9^ | 2 | RCT | General | None | None | Serious | Serious | 50 | 50 | RR = 0.33 (0.03, 2.16) | Low |

Abbreviations: IOP=intraocular pressure; BCVA=best corrected visual acuity; RCT=randomized controlled trial; MD=mean difference; RR=relative risk.

**Drug approval status in the United States, the European Union, and China**

None.

**Reference**

1. Arcieri ES, Paula JS, Jorge R, Barella KA, Arcieri RS, et al. Efficacy and safety of intravitreal bevacizumab in eyes with neovascular glaucoma undergoing Ahmed glaucoma valve implantation: 2-year follow-up. Acta Ophthalmologica. 2015;93(1):e1-e6.

2. Mahdy RA, Nada WM, Fawzy KM, Alnashar HY, Almosalamy SM. Efficacy of intravitreal bevacizumab with panretinal photocoagulation followed by ahmed valve implantation in neovascular glaucoma. Journal of Glaucoma. 2013;22(9):768-772.

3. Chang H, Qi F, Zhou ZJ, Li RX, Xu L, et al. The effect ofcombined trabeculectomy and panretinal photocoagulation with razumab on the visual function and inflammatory factors of aqueous humor in patients with neovascular glaucoma. Progress in Modern Biomedicine, 2021. 21(01): 174-177+196.

4. Chen T. [Ranibizumab combined with trabeculectomy in the treatment of neovascular glaucoma]. Journal of China Prescription Drug. 2021;19(03):105-107.

5. Gou JY. The application of conbercept in the treatment of diabetic retinopathy with stage Ⅰ and Ⅱ neovascular glaucoma. Chin J Ocul Fundus Dis, 2020. 36(10): 759-763.

6. He JL, Deng JQ, Deng XY, Song AZ, Ye J, Huang FP. [Research of intravitreal injection of ranibizumab combined with surgery in the treatment of neovascular glaucoma with vitreous hemorrhage]. Chin J Mod Drug Appl. 2020;14(18):197-199.

7. Li Li. Therapeutic effect of intravitreal injection of conbercept combined with trabeculectomy on neovascular glaucoma. Journal of Practical Preventing Blind, 2017. 12(04): 164-165+163.

8. Lin ZH, Wang ZL, Wang W, Nie CF, Zhang RR. Clinical observation of intravitreal injection of ranibizumab combined with argon laser therapy in the treatment of ischemic neovascular glaucoma. China Pharmacy, 2018. 29(10): 1380-1383.

9. Ma H. Effect of intravitreal injection of ranibizumab on neovascularization in patients with neovascular glaucoma after microsurgery. Chinese Journal of Rational Drug Use, 2019. 16(06): 59-61.

10. Xing MY, Yan ZJ. Clinical efficacy of ranibizumab intravitreal injection as adjuvant therapy for neovascular glaucoma. Medical Recapitulate, 2018. 24(05): p. 1037-1040.

11. Yan ZZ, Shi HH. Efficacy of intravitreal injection of razumab combined with scleral ciliary body photocoagulation in the treatment of neovascular glaucoma. China Journal of Modern Medicine, 2019. 29(14): 85-88.

12. Jiang WP, Lu SS, Jin Y. Clinical research of retinal laser photocoagulation and ranibizumab on the treatment of neovascular glaucoma. Int Eye Sci, 2015; 15(10): 1763-1765.

13. Inatani M, Higashide T, Matsushita K, Miki A, Ueki M, et al. Intravitreal Aflibercept in Japanese Patients with Neovascular Glaucoma: The VEGA Randomized Clinical Trial. Advances in Therapy. 2020.

## Q4 on anti-VEGF drugs for choroidal neovascularization

**Recommendation**

**PICO framework**

Patient: patients with choroidal neovascularization secondary to pathological myopia

Intervention: anti-VEGF drugs, including aflibercept, conbercept, ranibizumab, bevacizumab, brolucizumab

Comparison: photodynamic therapy (PDT) or placebo or PDT combination therapy

Outcome: all outcomes of interest were listed in the eTable below.

### Table S7. Summary of Findings and Certainty of Evidence Using GRADE Approach

| **Quality assessment** | | | | | | | | **Summary of findings** | | | |
| --- | --- | --- | --- | --- | --- | --- | --- | --- | --- | --- | --- |
|  |  |  |  |  |  |  |  | **Number of patients** | | **Effect size (95%CI)** | **Certainty of evidence** |
| **Outcomes** | **Number of included studies** | **Study design** | **Risk of bias** | **Inconsistency** | **Indirectness** | **Imprecision** | **Publication bias** | **Intervention group** | **Control group** |  |  |
| **Anti-VEGF drugs vs. placebo** | | | | | | | | | | | |
| BCVA^1^ | 1 | RCT | General | Not applicable | General | General | Serious | 90 | 31 | MD= -0.28 logMAR (-0.36, -0.20) | Low |
| CFT^1^ | 1 | RCT | General | Not applicable | General | General | Serious | 90 | 31 | MD=-66.80 (-114.87, -18.73) | Low |
| Severe ocular adverse events^1^ | 1 | RCT | General | Not applicable | General | General | Serious | 90 | 31 | RR = 2.46 (0.13, 46.36) | Low |
| Ocular adverse events^1^ | 1 | RCT | General | Not applicable | General | General | Serious | 90 | 31 | RR = 0.57 (0.28, 1.18) | Low |
| **Anti-VEGF drugs vs. photodynamic therapy** | | | | | | | | | | | |
| BCVA^2-5^ | 4 | RCT | General | General | General | General | None | 341 | 184 | MD= -0.14 logMAR (-0.17, -0.10) | High |
| CFT^3-5^ | 3 | RCT | General | General | General | General | None | 322 | 166 | MD= -44.32 (-59.85, -28.79) | High |
| Severe ocular adverse events^2-5^ | 4 | RCT | General | General | General | General | None | 341 | 184 | RR = 0.81 (0.11, 6.10) | High |
| Ocular adverse events^2-5^ | 4 | RCT | General | General | General | General | None | 341 | 184 | RR = 1.02 (0.77, 1.36) | High |
| **Anti-VEGF monotherapy vs. PDT combination therapy** | | | | | | | | | | | |
| BCVA^6,7^ | 2 | RCT | General | General | General | General | None | 37 | 37 | MD=0.07  (-0.00, 0.14) | High |
| CFT^7^ | 1 | RCT | General | Not applicable | General | General | Serious | 20 | 20 | MD=6.40 (-20.10,32.90) | Low |
| Severe ocular adverse events^6,7^ | 2 | RCT | General | General | General | General | None | 37 | 37 | None | High |
| Ocular adverse events^6,7^ | 2 | RCT | General | General | General | General | None | 37 | 37 | RR = 1.57 (0.77, 3.22) | High |

Abbreviations: BCVA=best corrected visual acuity; CFT= central foveal thickness, RCT=randomized controlled trial; MD=mean difference; RR=relative risk.

**Drug approval status in the United States, the European Union, and China**

**In the United States,** ranibizumab was approved for the treatment of myopic choroidal neovascularization (mCNV) in March 2018.

**In the European Union,** aflibercept was approved for the treatment of myopic choroidal neovascularization (mCNV) in July 2021. Ranibizumab was approved for use in choroidal neovascularization (CNV) in October 2020.

**In China,** conbercept was approved for the treatment of visual impairment caused by choroidal neovascularization (pmCNV) secondary to pathological myopia in May 2019.

**Reference**

1. Ikuno Y, Ohno-Matsui K, Wong TY, Korobelnik JF, Vitti R, et al. Intravitreal aflibercept injection in patients with myopic choroidal neovascularization: the MYRROR study. Ophthalmology. 2015; 122:1220-7.
2. Parodi MB, Iacono P, Papayannis A, Sheth S, Bandello F. Laser photocoagulation, photodynamic therapy, and intravitreal bevacizumab for the treatment of juxtafoveal choroidal neovascularization secondary to pathologic myopia. Arch Ophthalmol. 2010; 128:437-42.
3. Ruiz-Moreno JM, López-Gálvez MI, Montero Moreno JA, Pastor Jimeno JC. Intravitreal bevacizumab in myopic neovascular membranes: 24-month results. Ophthalmology. 2013; 120:1510-1.e1.
4. Wolf S, Balciuniene VJ, Laganovska G, Menchini U, Ohno-Matsui K, et al. RADIANCE: a randomized controlled study of ranibizumab in patients with choroidal neovascularization secondary to pathologic myopia. Ophthalmology. 2014; 121:682-92.e2.
5. Chen Y, Sharma T, Li X, Song Y, Chang Q, et al. Ranibizumab versus verteporfin photodynamic therapy in Asian patients with myopic choroidal neovascularization: BRILLIANCE, a 12-month, randomized, double-masked study. Retina. 2019; 39:1985-1994.
6. Saviano S, Piermarocchi R, Leon PE, Mangogna A, Zanei A, et al. Combined therapy with bevacizumab and photodynamic therapy for myopic choroidal neovascularization: a one-year follow-up controlled study. Int J Ophthalmol. 2014; 7:335-9.
7. Rinaldi M, Semeraro F, Chiosi F, Russo A, Romano MR, et al. Reduced-fluence verteporfin photodynamic therapy plus ranibizumab for choroidal neovascularization in pathologic myopia. Graefes Arch Clin Exp Ophthalmol. 2017; 255:529-539.

## Q5 on anti-VEGF drugs for macular edema secondary to retinal vein occlusion

**Recommendation 1 & 2**

**PICO framework**

Patient: patients with macular edema secondary to retinal vein occlusion (RVO-ME)

Intervention: anti-VEGF drugs, including aflibercept, conbercept, ranibizumab, bevacizumab, brolucizumab

Comparison: placebo, panretinal laser photocoagulation, local injection of glucocorticoid (dexamethasone intravitreal implant, triamcinolone acetonide)

Outcome: all outcomes of interest were listed in the eTable below

### Table S8. Summary of Findings and Certainty of Evidence Using GRADE Approach

| **Quality assessment** | | | | | | | | **Summary of findings** | | | |
| --- | --- | --- | --- | --- | --- | --- | --- | --- | --- | --- | --- |
|  |  |  |  |  |  |  |  | **Number of patients** | | **Effect size (95%CI)** | **Certainty of evidence** |
| **Outcome** | **Number of included studies** | **Study design** | **Risk of bias** | **Inconsistency** | **Indirectness** | **Imprecision** | **Publication bias** | **Intervention group** | **Control group** |  |  |
| **Anti-VEGF drugs vs. placebo** | | | | | | | | | | | |
| BCVA at 6 months^1-5^ | 5 | RCT | General | Serious | General | General | None | 847 | 454 | MD = 12.69 letters (8.10, 17.28) | Moderate |
| Visual acuity improvement ≥15 letters at 6 months^1-5^ | 5 | RCT | General | Serious | General | General | None | 847 | 454 | RR = 2.37 (1.70, 3.30) | Moderate |
| Vision loss ≥15 letters at 6 months^2-4^ | 3 | RCT | General | General | General | General | None | 567 | 297 | RR = 0.13 (0.07, 0.26) | High |
| CMT at 6 months^2,3,4,14,15,16^ | 6 | RCT | Serious | Serious | General | General | None | 834 | 564 | MD = 153.61 (256.78, 50.43) | Low |
| NEIVFQ-25 changes at 6 months^2,4,5^ | 3 | RCT | General | General | General | General | None | 551 | 288 | MD = 5.01 (3.23, 6.79) | High |
| Proportion of patients with elevated intraocular pressure at 6 months^2,3,5,14,15,16^ | 6 | RCT | Serious | General | General | General | None | 675 | 503 | RR = 2.37 (1.17, 4.81) | Moderate |
| Ocular adverse events at 6 months^3,4^ | 2 | RCT | General | Serious | General | General | None | 297 | 162 | RR = 1.90 (0.51, 7.13) | Moderate |
| Non-ocular severe adverse events at 6 months^2-4^ | 3 | RCT | General | General | General | General | None | 564 | 296 | RR = 0.94 (0.42, 2.15) | High |
| BCVA at 12 months^2,9,10,11^ | 4 | RCT | General | General | General | General | None | 669 | 364 | MD = 7.14 letters (5.43, 8.85) | High |
| Visual acuity improvement ≥15 letters at 12 months^2,9,10,11^ | 4 | RCT | General | General | General | General | None | 669 | 364 | MD = 1.59 (1.35, 1.86) | High |
| Vision loss ≥15 letters at 12 months^2,9,10,11^ | 4 | RCT | General | General | General | General | None | 669 | 364 | MD = 0.28 (0.16, 0.49) | High |
| CMT at 12 months^2,10^ | 2 | RCT | General | Serious | General | General | None | 304 | 166 | MD = -150.06 (-465.51, 165.38) | Moderate |
| NEIVFQ-25 changes at 12 months^2,10^ | 2 | RCT | General | General | General | General | None | 304 | 166 | MD = 6.35 (3.97, 8.73) | High |
| Non-ocular severe adverse events at 12 months^10,11^ | 2 | RCT | General | General | General | General | None | 376 | 204 | RR = 0.87 (0.36, 2.12) | High |
| **Anti-VEGF drugs vs. glucocorticoid** | | | | | | | | | | | |
| BCVA at 6 months^7,13^ | 2 | RCT | General | Serious | General | General | None | 250 | 237 | MD = 12.68 letters (3.37, 21.98) | Moderate |
| Visual acuity improvement ≥15 letters at 6 months^7,13^ | 2 | RCT | General | Serious | General | General | None | 250 | 237 | RR = 4.04 (1.72, 9.48) | Moderate |
| CMT at 6 months^7,13^ | 2 | RCT | General | Serious | General | General | None | 250 | 237 | MD = 158.53 (245.96, 71.09) | Moderate |
| Proportion of patients with elevated intraocular pressure at 6 months^7,13^ | 2 | RCT | General | General | General | General | None | 250 | 237 | RR = 0.12 (0.06, 0.25) | High |
| Non-ocular severe adverse events at 6 months^7,13^ | 2 | RCT | General | Serious | General | General | None | 250 | 237 | RR = 0.89 (0.44, 1.80) | Moderate |
| Incidence of ocular adverse events at 6 months^7,13^ | 2 | RCT | General | Serious | General | General | None | 250 | 237 | RR = 0.50 (0.31, 0.79) | Moderate |
| **Anti-VEGF drugs vs. panretinal laser photocoagulation** | | | | | | | | | | | |
| BCVA at 6 months^1,12^ | 2 | RCT | General | General | General | General | None | 274 | 184 | MD = 9.41 letters (6.95, 11.86) | High |
| Visual acuity improvement ≥15 letters at 6 months^1,12^ | 2 | RCT | General | General | General | General | None | 274 | 184 | RR = 2.49 (1.65, 3.75) | High |
| CMT at 6 months^1,12,15^ | 3 | RCT | Serious | Serious | General | General | None | 330 | 240 | MD = 101.01 (179.75, 22.27) | Low |
| Proportion of patients with elevated intraocular pressure at 6 months^1,15^ | 2 | RCT | Serious | Serious | General | General | None | 239 | 148 | RR = 4.56 (0.53, 39.14) | Low |
| Non-ocular severe adverse events at 6 months^1,12^ | 2 | RCT | General | General | General | General | None | 274 | 184 | RR = 1.29 (0.58, 2.89) | High |
| Ocular adverse events at 6 months^1,12^ | 2 | RCT | General | General | General | General | None | 274 | 184 | RR = 0.25 (0.05, 1.30) | High |

Abbreviations: BCVA=best corrected visual acuity; CMT= central macular thickness, RCT=randomized controlled trial; MD=mean difference; RR=relative risk.

**Drug approval status in the United States, the European Union, and China**

**In the United States,** ranibizumab was approved for the treatment of RVO-ME in June 2010. Aflibercept was approved for the treatment of central RVO-ME, RVO-ME in September 2012, October 2014 respectively.

**In the European Union,** ranibizumab was approved for the treatment of RVO-ME in May 2011. Aflibercept was approved for the treatment of central RVO-ME, branch RVO-ME in August 2013, February 2015 respectively.

**In China,** ranibizumab was approved for the treatment of RVO-ME in May 2018.

**Reference:**

1. Tadayoni R, Waldstein SM, Boscia F, et al. Sustained benefits of ranibizumab with or without laser in branch retinal vein occlusion: 24-month results of the BRIGHTER study. Ophthalmology. 2017; 124(12):1778-1787.
2. Wei W, Weisberger A, Zhu L, et al. Efficacy and Safety of Ranibizumab in Asian Patients with Branch Retinal VeinOcclusion: Results from the Randomized BLOSSOM Study, Ophthalmology Retina. 2020; 4(1): 57-66.
3. David B, Jeffrey H, David MB, et al. Vascular Endothelial Growth Factor Trap-Eye for Macular Edema Secondary to Central Retinal Vein Occlusion: Six-Month Results of the Phase 3 COPERNICUS Study. Ophthalmology. 2012; 119(5): 1024-1032.
4. David MB, Peter AC, Rishi PS, et al. Ranibizumab for Macular Edema following Central Retinal Vein Occlusion Six-Month Primary End Point Results of a Phase III Study. Ophthalmology. 2010; 117:1124-1133.
5. Holz FG, Roider J, Ogura Y, et al. VEGF Trap-Eye for macular oedema secondary to central retinal vein occlusion: 6-month results of the phase III GALILEO study. British Journal of Ophthalmology. 2013;97:278-284.
6. Bandello F, Augustin A, Tufail A, et al. A 12-month, multicenter, parallel group comparison of dexamethasone intravitreal implant versus ranibizumab in branch retinal vein occlusion. European Journal of Ophthalmology. 2018;28(6):697-705.
7. Hattenbach LO, Feltgen N, Bertelmann T, et al. Head-to-head comparison of ranibizumab PRN versus single-dose dexamethasone for branch retinal vein occlusion (COMRADE-B). Acta Ophthalmologica. 2018; 96(1):e10-8.
8. Campochiaro PA, Clark WL, Boyer DS, et al. Intravitreal aflibercept for macular edema following branch retinal vein occlusion: the 24-week results of the VIBRANT study. Ophthalmology. 2015;122(3):538-544.
9. Jean-Francois K, Frank H, Johann R, et al. Intravitreal Aflibercept Injection for Macular Edema Resulting from Central Retinal Vein Occlusion: One-Year Results of the Phase 3 GALILEO Study. Ophthalmology: Journal of The American Academy of Ophthalmology. 2014; 121 (1): 202-208.
10. David MB, Jeffrey SH, Lloyd C, et al. Intravitreal Aflibercept Injection for Macular Edema Secondary to Central Retinal Vein Occlusion: 1-Year Results From the Phase 3 COPERNICUS Study. Am J Ophthalmol. 2013; 155: 429-437.
11. Peter AC, David MB, Carl CA, et al. Sustained Benefits from Ranibizumab for Macular Edema following Central Retinal Vein Occlusion: Twelve-Month Outcomes of a Phase III Study. Ophthalmology. 2011;118:2041-2049.
12. Lloyd C, David SB, Jeffrey SH, et al. Intravitreal Aflibercept for Macular Edema Following Branch Retinal Vein Occlusion: 52-Week Results of the VIBRANT Study. Ophthalmology. 2016; 123(10):e60.
13. Hans H, Nicolas F, Claudia W, et al. Clinical Efficacy and Safety of Ranibizumab Versus Dexamethasone for Central Retinal Vein Occlusion (COMRADE C): A European Label Study. Am J Ophthalmol. 2016 ;169:258-267.
14. Duan YP, Hu Y, Liu Y, et al. The curative effect of intravitreal injection of Conbercept combined with triamcinolone ace- tonide in the treatment of macular edema secondary to retinal vein occlusion. Practical Journal of Clinical Medicine. 2019;16 (3): 127-130.
15. Guo CL, Chen DM, Wang SL.Comparison of therapeutic effect of different methods on treatment of macular edema secondary to branch retinal vein occlusion. Chin J New Drugs Clin Rem. 2018; 37(10): 586-590.
16. Jiang XG, Han XD, Yang M. Effect of Compaq-Pac intravitreal injection with laser photocoagulation in the treatment of retinal vein occlusive macular cystic edema and its effect on visual acuity. Practical Journal of Clinical Medicine. 2018; 15(4): 27-29.

## Q6 on anti-VEGF drugs for Coats' disease

**Recommendation**

**PICO framework**

Patient: patients with Coats' disease at all stages

Intervention: anti-VEGF drugs or anti-VEGF drugs combined with ablation therapy, among which anti-VEGF drugs include aflibercept, conbercept, ranibizumab, bevacizumab and brolucizumab.

Comparison: ablation therapy or placebo

Outcome: all outcomes of interest were listed in the eTable below.

### Table S9. Summary of Findings and Certainty of Evidence Using GRADE-CERQual Approach

| Outcomes | Study design | Methodological limitations^a^ | Coherence^b^ | Relevance^c^ | Adequacy^d^ | Summary of findings | CERQual rating |
| --- | --- | --- | --- | --- | --- | --- | --- |
| Visual acuity^1-4, 5-17, 19-24^ | 22 case series | Moderate | High | High | Moderate, involving five countries | Positive influence in visual acuity after anti-VEGF treatment in 21 studies. | Low |
| CMT^1,8,11,13^ | 4 case series | Moderate | Moderate | High | Low, involving 2 countries | One study showed no significant change in CMT before and after treatment, and three studies showed thinning of CMT after anti-VEGF drug therapy. | Very low |
| Retinal reattachment^7.9,15,16,19,23^ | 5 case series | Moderate | High | High | Low, involving 3 countries | Among 57 patients, 42.11% achieved in complete retinal reattachment, 38.60% were succeeded in partial retinal reattachment, and 19.29% failed in retinal reattachment. | Very low |
| Adverse events^1-6,8,10-15,17-24^ | 21 case series | Moderate | Moderate | High | Moderate, involving 6 countries | Fibrosis change was the most common adverse event, followed by cataract formation, traction retinal detachment, neovascular glaucoma and vitreous hemorrhage, without systemic adverse events | Low |

Abbreviations: CMT=central macular thickness.

Explanation:

a. Methodological limitations: the extent to which there are concerns about the design or conduct of the included studies supporting the review finding;

b. Coherence: an assessment of how clear and cogent the fit is between the data from the included studies the review finding;

c. Relevance: the extent to which the body of data from the included studies supporting the review finding is applicable to the context specified in the review question;

d. Adequacy: the degree of richness and quantity of data supporting the review finding;

The overall quality of qualitative evidence for each outcome of the other clinical questions was graded considering above domains using GRADE-CERQual approach

**Drug approval status in the United States, the European Union, and China**

None.

**Reference**

1. Cennamo G, Montorio D, Comune C, et al. Optical Coherence Tomography Angiography Findings After Intravitreal Ranibizumab in Patients With Coats Disease. Frontiers in Medicine (Lausanne). 2021; 21: 615015.
2. Kang HG, Kim JD, Choi EY, et al. Clinical features and prognostic factors in 71 eyes over 20 years from patients with Coats' disease in Korea. Scientific Reports. 2021; 11: 6124.
3. Nowara M, Fouad YA, Aziz IA, et al. Experience with Intravitreal Ranibizumab as an Adjunct to Ablation Therapy in Eyes with Exudative Coats' Disease. Clinical Ophthalmology. 2021; 15: 367-373.
4. Jiang L, Qin B, Luo XL, et al. Three-year follow-up of Coats disease treated with conbercept and 532-nm laser photocoagulation. World Journal of Clinical Cases. 2020; 8: 6243-6251.
5. Liang TY, Peng J, Zhang Q, et al. Management of stage 3B Coats disease: presentation of a combined treatment modality and long-term follow-up. Graefes Archive for Clinical and Experimental Ophthalmology. 2020; 258: 2031-2038.
6. Zhang L, Ke Y, Wang W, et al. The efficacy of conbercept or ranibizumab intravitreal injection combined with laser therapy for Coats' disease. Graefes Archive for Clinical and Experimental Ophthalmology. 2018; 256: 1339-1346.
7. Huang YX, Nie XG, Fan DS, et al. Efficacy of intravitreal injection of anti-VEGF drugs combination with other methods for Coats disease. International Eye Science. 2017; 17: 1783-1785.
8. Jing L, Li J, Peng W. Clinical analysis of intravitreal inject ion of Conbercept combined with 532- laser treating Coats disease in adulthood. International Eye Science. 2017; 17: 1356-1358.
9. Li S, Deng GD, Liu JH, et al. The effects of a treatment combination of anti-VEGF injections, laser coagulation and cryotherapy on patients with type 3 Coat's disease. BMC Ophthalmology. 2017; 17: 76.
10. Li SF, Deng GD, Liu JH, et al. Subretinal fluid drainage combined with intravitreal injection of anti-vascular endothelial growth factor in treatment of severe exudative retinal detachment Coats disease. Recent Advances in Ophthalmology. 2017; 37: 569-571.
11. Mao XB, You ZP, Peng L, et al. Intravitreal lucentis injections combined with laser photocoagulation for Coats disease. Recent Advances in Ophthalmology. 2017; 37: 259-262.
12. Bhat V, D'Souza P, Shah PK, et al. Risk of Tractional Retinal Detachment Following Intravitreal Bevacizumab Along with Subretinal Fluid Drainage and Cryotherapy for Stage 3B Coats' Disease. Middle East African Journal of Ophthalmology. 2016; 23: 208-211.
13. Park S, Cho HJ, Lee DW, et al. Intravitreal bevacizumab injections combined with laser photocoagulation for adult-onset Coats' disease. Graefes Archive for Clinical and Experimental Ophthalmology. 2016; 254: 1511-1517.
14. Yang Q, Wei W, Shi X, et al. Successful use of intravitreal ranibizumab injection and combined treatment in the management of Coats' disease. Acta Ophthalmologica. 2016; 94: 401-406.
15. Chen H, Chen YX, Han R. Outcomes of adjuvant intravitreal anti-vascular endothelial growth factor therapy in advanced Coats disease. Chinese Journal of Ocular Fundus Diseases. 2015; 31: 252-255.
16. Wang S, Song ZX. Ranibizumab combined with vitrectomy and photocoagulation in the treatment of vitreous hemorrhage and retinal detachment caused by Coats. Journal of Henan Medical College. 2015; 27: 122-124.
17. Gaillard MC, Mataftsi A, Balmer A, et al. ranibizumab in the management of advanced Coats disease Stages 3B and 4: long-term outcomes. Retina. 2014; 34: 2275-2281.
18. Villegas VM, Gold AS, Berrocal AM, et al. Advanced Coats' disease treated with intravitreal bevacizumab combined with laser vascular ablation. Clinical Ophthalmology. 2014; 8: 973-976.
19. Yang Q, Wei WB, Liu YM. Intravitreal injection of ranibizumab and combined treatment for severe Coats disease. Chinese Journal of Ocular Fundus Diseases. 2014; 30:462-465.
20. Zheng XX, Jiang YR. The effect of intravitreal bevacizumab injection as the initial treatment for Coats' disease. Graefes Archive for Clinical and Experimental Ophthalmology. 2014; 252: 35-42.
21. Ramasubramanian A, Shields CL. Bevacizumab for Coats' disease with exudative retinal detachment and risk of vitreoretinal traction. British Journal of Ophthalmology. 2012; 96: 356-359.
22. Goel N, Kumar V, Seth A, et al. Role of intravitreal bevacizumab in adult onset Coats' disease. International Ophthalmology. 2011; 31:183-190.
23. Kaul S, Uparkar M, Mody K, et al. Intravitreal anti-vascular endothelial growth factor agents as an adjunct in the management of Coats' disease in children. Indian Journal of Ophthalmology. 2010; 58: 76-78.
24. Lin CJ, Hwang JF, Chen YT, et al. The effect of intravitreal bevacizumab in the treatment of Coats disease in children. Retina. 2010; 30: 617-622.

## Q7 on local ocular triamcinolone acetonide injection for uveitis

**Recommendation**

**PICO framework**

Patient: patients with uveitis

Intervention: local application of triamcinolone acetonide or triamcinolone acetonide in combination with other glucocorticoids

Comparison: local use of other glucocorticoid or placebo

Outcome: all outcomes of interest were listed in the eTable below

### Table S10. Summary of Findings and Certainty of Evidence Using GRADE-CERQual Approach approach

| **Outcomes** | **Study design** | **Methodological limitations** | **Coherence** | **Relevance** | **Adequacy** | **Summary of findings** | **CERQual rating** |
| --- | --- | --- | --- | --- | --- | --- | --- |
| Visual acuity^1-16^ | 10 case series  3 case-controls  1 cohort study  2 RCTs | Moderate | High | High | High, involving 10 countries | Two RCTs showed that triamcinolone acetonide group significantly improved early postoperative visual acuity compared with control group.  A cohort study reported no significant improvement in visual acuity in triamcinolone compared with methylprednisolone.  These results were reported in three case-control studies. One study reported that PSTA group had no significant improvement in visual acuity compared with DEX implant group. One study reported that the visual acuity recovery rate (visual acuity > 0.5) of triamcinolone acetonide low-dose group was significantly higher than that of high-dose group. One study reported that the proportion of corrected visual acuity > 0.5 in triamcinolone acetonide group was significantly higher than that in control group.  The results were reported in 10 case series studies. Two of the studies reported no difference in visual acuity before and after treatment, and the remaining eight studies reported significant improvement in visual acuity before and after treatment. | Moderate |
| IOP^1-4,6,10,12,13,15,16^ | 6 case series  2 case-controls  2 RCTs | Moderate | Moderate | High | High, involving 5 countries | The results were reported in two RCTs and two case-control studies. One study reported that IOP in the low-dose triamcinolone acetonide group was significantly lower than that in the high-dose group after treatment. Three studies reported no significant difference in postoperative IOP between groups after treatment.  The results were reported in six case series studies. There were 4 reports that IOP increased slightly after treatment. There was 1 report of missing IOP data after treatment. One report showed a significant decrease in IOP after treatment. | Moderate |
| Corneal endothelial cell count^12,13,15,16^ | 2 case-control study  2 RCTs | Moderate | High | High | Low, involving 1 country | Two RCT and two case-control studies reported no significant difference in corneal endothelial cell counts between groups after treatment. | Moderate |
| Anterior chamber inflammation^13,16^ | 1 case-control  1 RCT | Moderate | High | High | High, involving 1 country | Results of one RCT study and one case-control study showed that postoperative anterior chamber inflammation in triamcinolone acetonide group was significantly lower than that in control group. | High |

Abbreviations: BCVA=best corrected visual acuity; IOP= intraocular pressure; RCT=randomized controlled trial; PSTA= posterior sub-Tenon injection of triamcinolone acetonide; DEX= Dexamethasone.

**Drug approval status in the United States, the European Union, and China**

**In the United States,** triamcinolone acetonide suprachoroidal injectable suspension was approved for the treatment of macular edema associated with uveitis in October 2021.

**In the European Union,** triamcinolone acetonide was granted orphan designation for the treatment of non-infectious uveitis in May 2015.

**In China,** triamcinolone acetonide injection (Kenacort-A) was approved for the treatment of uveitis.

**Reference**

1. Andjelic G, Jovanović S, Pesic S, Mitrasevic M, Stojanovic J, Radotic F, Todorovic D, Petrovic N. The efficacy of a posterior sub-tenon’s capsule triamcinolone injection in patients with non-infectious intermediate uveitis and posterior uveitis. Serbian Journal of Experimental and Clinical Research. 2017;18(2), 151-156.
2. Goldstein DA, Do D, Noronha G, Kissner JM, Srivastava SK, Nguyen QD. Suprachoroidal Corticosteroid Administration: A Novel Route for Local Treatment of Noninfectious Uveitis. Transl Vis Sci Technol. 2016; 5(6), 14.
3. Henry CR, Shah M, Barakat MR, Dayani P, Wang RC, Khurana RN, Ciulla T. Suprachoroidal CLS-TA for non-infectious uveitis: an open-label, safety trial (AZALEA). Br J Ophthalmol. 2021; 318019.
4. Helm CJ., Holland GN. The effects of posterior subtenon injection of triamcinolone acetonide in patients with intermediate uveitis. Am J Ophthalmol. 1995;120(1), 55-64.
5. Roesel M, Gutfleisch M, Heinz C, Heimes B, Zurek-Imhoff B, Heiligenhaus A. Orbital floor triamcinolone acetonide injections for the management of active non-infectious uveitis. Eye (Lond). 2009;23(4), 910-914.
6. Alkawas AA, Hamdy AM, Shahien EA. Intraoperative intravitreal injection of triamcinolone acetonide for cataract extraction in patients with uveitis. Ocul Immunol Inflamm. 2010;18(5), 402-407.
7. Narciss O, Ainsley M, Howe SK, Victor M, et al. Intraoperative use of intravitreal triamcinolone in uveitic eyes having cataract surgery: pilot study. J Cataract Refract Surg. 2007; 33(7), 1278-1283.
8. Park UC, Park JH, Yu HG. Long-term outcome of intravitreal triamcinolone acetonide injection for the treatment of uveitis attacks in Behçet disease. Ocul Immunol Inflamm. 2014;22(1), 27-33.
9. Tuncer S, Yilmaz S, Urgancioglu M, Tugal-Tutkun I. Results of intravitreal triamcinolone acetonide (IVTA) injection for the treatment of panuveitis attacks in patients with Behçet disease. J Ocul Pharmacol Ther. 2007;23(4), 395-401.
10. Keorochana N, Kunasuntiwarakul S, Treesit I, Choontanom R. The efficacy of preoperative posterior subtenon injection of triamcinolone acetonide in noninfectious uveitic patients with secondary glaucoma undergoing trabeculectomy. Clin Ophthalmol.2017;11, 2057-2063.
11. Errera MH, Westcott M, Benesty J, Falah S, Smadja J, Orès R, Sahel JA. A Comparison of the Dexamethasone Implant (Ozurdex®) and Inferior Fornix-Based Sub-Tenon Triamcinolone Acetonide for Treatment of Inflammatory Ocular Diseases. Ocul Immunol Inflamm.2019;27(2), 319-329.
12. Wang HY, Zhu WH, Liu XF. [Efficacy Evaluation of Intravitreal Injection of Low-Dose Triamcinolone Acetonide in the Treatment of Patients with Uveitis After Cataract Surgery]. China Pharmaceuticals. 2021;30(06), 83-85.
13. Li R, Yuan HM, Liu ZC, Li Y. [Clinical effect of adjuvant therapy with glucocorticoid in surgical treatment of uveitis complicated cataract]. International Eye Science. 2016;16(12), 2254-2257.
14. Ferrante P, Ramsey A, Bunce C, Lightman S. Clinical trial to compare efficacy and side-effects of injection of posterior sub-Tenon triamcinolone versus orbital floor methylprednisolone in the management of posterior uveitis. Clin Exp Ophthalmol. 2004;32(6), 563-568.
15. Xu B, Dong N, Xiao L, Chu LQ, Xu JM, Wang BS. [Intracameral different dosage triamcinolone acetonide for anterior uveitis after cataract combined with anti-glaucoma surgery]. Recent Advances in Ophthalmology. 2012; 32(06):566-569.
16. Liu KX, Zhang GW, Liu XM, Li YH. [Observation of anterior chamber triamcinolone acetonide injection after anterior uveitis complicated cataract surgery]. Journal of Clinical Ophthalmology. 2013;21(03), 208-210.

## Q8 on local ocular triamcinolone acetonide injection for macular edema

**Recommendation**

**PICO framework**

Patient: patients with macular edema due to various causes (including diabetes, uveitis, retinitis pigmentosa, post-surgery, retinal vein occlusion)

Intervention: triamcinolone acetonide (intravitreal injection, retrocapsular injection, peribulbar injection)

Comparison: placebo, anti-VEGF drugs

Outcome: all outcomes of interest were listed in the eTable below.

### Table S11. Summary of Findings and Certainty of Evidence Using GRADE Approach

| **Quality assessment** | | | | | | | | **Summary of findings** | | | |
| --- | --- | --- | --- | --- | --- | --- | --- | --- | --- | --- | --- |
|  |  |  |  |  |  |  |  | **Number of patients** | | **Effect size (95%CI)** | **Certainty of evidence** |
| **Outcomes** | **Number of included studies** | **Study design** | **Risk of bias** | **Inconsistency** | **Indirectness** | **Imprecision** | **Publication bias** | **Intervention group** | **Control group** |  |  |
| **Triamcinolone acetonide vs. placebo** | | | | | | | | | | | |
| BCVA at 3 months^6,9,15,19,28,30,33^ | 7 | RCT | Serious | Serious | General | General | None | 231 | 241 | MD = -0.02 (-0.08, 0.04) | Low |
| CMT at 3 months^5,6,9,10,11,14,15,19,20,21,28,30,33^ | 13 | RCT | Serious | General | General | General | None | 426 | 431 | MD = -36.95 (-48.92, -24.98) | Moderate |
| IOP at 3 months^5,9,10,19,20,30^ | 6 | RCT | Serious | General | General | General | None | 213 | 211 | MD = 0.72 (0.21, 1.22) | Moderate |
| Incidence of IOH at 3 months^6,10,15,33^ | 4 | RCT | Serious | General | General | General | None | 121 | 129 | RR = 9.55 (1.79, 50.85) | Moderate |
| BCVA at 6 months^6,9,19,25,28,30,33^ | 7 | RCT | General | Serious | General | General | None | 240 | 249 | MD = -0.01 (-0.08, 0.05) | High |
| CMT at 6 months^1,2,5,6,9,11,14,18,19,21,25,28,30,33^ | 14 | RCT | Serious | Serious | General | General | None | 537 | 509 | MD = -46.38 (-69.40, -23.36) | Low |
| IOP at 6 months^5,19,30^ | 3 | RCT | Serious | Serious | General | General | None | 106 | 102 | MD = 0.16 (-1.61, 1.93) | Low |
| Incidence of IOH at 6 months^1,2,6,14,18,21,33^ | 7 | RCT | Serious | General | General | General | None | 277 | 253 | RR = 1.51 (0.70, 3.25) | Moderate |
| BCVA at 12 months^25,26^ | 2 | RCT | General | General | General | General | None | 75 | 84 | MD = 0.10 (0.01, 0.19) | High |
| CMT at 12 months^3,25,26^ | 3 | RCT | General | Serious | General | General | None | 261 | 377 | MD = 3.70 (-37.66, 45.06) | High |
| Incidence of IOH at 12 months^3,25^ | 2 | RCT | General | General | General | General | None | 222 | 332 | RR = 7.64 (4.60, 12.68) | High |
| **Triamcinolone acetonide vs. anti-VEGF drugs** | | | | | | | | | | | |
| BCVA at 3 months^9,12,22,23,24,27,29,32^ | 8 | RCT | General | General | General | General | None | 187 | 192 | MD = 0.04 (-0.01, 0.09) | High |
| CMT at 3 months^4,9,12,17,22,23,24,27,29,31,32^ | 11 | RCT | General | General | General | General | None | 264 | 269 | MD = 0.45 (-1.32, 2.23) | High |
| IOP at 3 months^4,9,22,27,29,31^ | 6 | RCT | General | General | General | General | None | 166 | 168 | MD = 0.75 (-0.09, 1.58) | High |
| Incidence of IOH at 3 months^22,24,27^ | 3 | RCT | General | General | General | General | None | 100 | 102 | RR = 4.45 (1.32, 14.98) | High |
| BCVA at 6 months^8,9,12,22,23,27,29,32^ | 8 | RCT | General | Serious | General | General | None | 199 | 204 | MD = 0.03 (-0.1, 0.15) | High |
| CMT at 6 months^4,8,9,12,17,22,23,27,29,32^ | 10 | RCT | General | General | General | General | None | 246 | 251 | MD = 2.01 (1.27, 2.75) | High |
| IOP at 6 months^4,9,22,27,29^ | 5 | RCT | Serious | General | General | General | None | 136 | 138 | MD = 1.16 (-0.32, 2.64) | Moderate |
| Incidence of IOH at 6 months^12,22,23,27^ | 4 | RCT | General | General | General | General | None | 109 | 112 | RR = 4.13 (1.44, 11.89) | High |
| BCVA at 12 months^8,27,32^ | 3 | RCT | General | General | General | General | None | 84 | 86 | MD = 0.02 (-0.07, 0.12) | High |
| CMT at 12 months^3,8,17,27,32^ | 5 | RCT | General | General | General | General | None | 294 | 297 | MD = -1.00 (-1.77, -0.23) | High |
| IOP at 12 months^8,27^ | 2 | RCT | General | Serious | General | General | None | 69 | 71 | MD = -0.17 (-2.81, 2.48) | Moderate |
| Incidence of IOH at 12 months^3,8,17,27^ | 4 | RCT | General | General | General | General | None | 279 | 282 | RR = 7.46 (4.30, 12.96) | High |

Abbreviations: BCVA=best corrected visual acuity; CMT= central macular thickness; IOP= intraocular pressure; IOH= intraocular hypertension; RCT=randomized controlled trial; MD=mean difference; RR=relative risk.

**Drug approval status in the United States, the European Union, and China**

**In the United States,** triamcinolone acetonide injectable suspension was approved for the treatment of macular edema associated with uveitis in October 2021.

**In the European Union,** none.

**In China,** none.

**Reference**

1. Bo L, Han L. Clinical Observation of Efficacy of Triamcinolone Acetonide Combined with Laser Photocoagulation in the Treatment of Diabetic Macular Edema Patients and Their Influence on Life Quality. Chin J Pharmacoepidemiol. 2017; 26(7): 446-449.
2. Cui S, Hu Y，Li Z. Observation on Efficacy of Retrobulbar Injection of Triamcinolone Acetonide Combined with Laser Photocoagulation in Treatment of Retinal Vein Obstruction Secondary to Cystoid Macular Edema.Evaluation and Analysis of Drug-Use in Hospitals of China. 2016; 16(10): 1367-1369.
3. Michael J. Elman, Lloyd Paul Aiello, Roy W. Beck, et al. Randomized Trial Evaluating Ranibizumab Plus Prompt or Deferred Laser or Triamcinolone Plus Prompt Laser for Diabetic Macular Edema. Ophthalmology 2010;117:1064-1077.
4. Hu XM, Hu M, Huang ZJ, et al. The effects of intravitreal injection of ranibizumab in treatment of macular edema secondary to central retinal vein occlusion. Ophthalmol CHN. 2017; 26(2): 116-119.
5. Li SY，Miao L，Chen H, et al. Efficacy and safety of combination treatment with triamcinolone acetonide retrobulbar injection and panretinal photocoagulation in diabetic macular edema. Journal of Jilin University (Medicine Edition). 2014; 40(6):1289-1292.
6. Li TT, Niu TT, Wang HL. lntravitreal injection with ranibizumab combined with triamcinolone acetonide sub - Tenon injection for macular edema due to CRVO. Int Eye Sci. 2015; 15(1): 98-100.
7. DSC Lam，CKM Chan，S Mohamed，et al. Intravitreal Triamcinolone plus Sequential Grid Laser versus Triamcinolone or Laser Alone for Treating Diabetic Macular Edema: Six-Month Outcomes.Ophthalmology. 2007;114:2162-2167.
8. Ji Won Lim, Hyo Kyoung Lee, Min Cheol Shin. Comparison of Intravitreal Bevacizumab Alone or Combined with Triamcinolone versus Triamcinolone in Diabetic Macular Edema: A Randomized Clinical Trial. Ophthalmologica. 2012;227:100-106.
9. Liu JB, Zhang H. Phacoemulsification combined with intravitreal ranibizumab or triamcinolone acetonide injection for the treatment of cataract accompanied by diabetic macular edema. Journal of Otolaryngology and Ophthalmology of Shandong University. 2019; 33(2): 99-104.
10. Luo CP, Tian SC, Li HL, et al. Triamcinolone acetonide vitreous injection associated with macular grid photocoagulation for macular edema in diabetic retinopathy. Recent Advances in Ophthalmology. 2010; 30(9): 851-853.
11. Lv XD, Gong LL, Chai JQ. Effect of Intravitreal Injection of Triamcinolone Acetonide Combined with Laser Photocoagulation in Treatment of 32 Cases of Diabetic Macular Edema. Herald of Medicine. 2019; 38(1): 62-65.
12. Pan ZJ, Zhang ZH, Fan FH, et al. Clinical effect and cost analysis of panretinal photocoagulation combined with Ranibizumab or triamcinolone acetonide for diabetic macular edema. Int Eye Sci. 2018; 18(4): 682-685.
13. The SCORE Study Research Group. A Randomized Trial Comparing the Efficacy and Safety of Intravitreal Triamcinolone With Standard Care to Treat Vision Loss Associated With Macular Edema Secondary to Branch Retinal Vein Occlusion: The Standard Care vs Corticosteroid for Retinal Vein Occlusion (SCORE) Study Report 6. Arch Ophthalmol. 2009;127(9):1115-1128.
14. Sheng YJ, Wang Y, Shu XW. Intravitreal triamcinolone acetonide and laser photocoagulation for diabetic macular edema. Journal of Otolaryngology and Ophthalmology of Shandong University. 2008; 22(6): 541-543.
15. Soheilian M, Ramezani A, Bijanzadeh B, et al. Intravitreal bevacizumab (avastin) injection alone or combined with triamcinolone versus macular photocoagulation as primary treatment of diabetic macular edema. Retina. 2007; 27(9):1187-95.
16. Jennifer E. Thorne, Elizabeth A. Sugar, Janet T, et al. Periocular Triamcinolone vs. Intravitreal Triamcinolone vs. Intravitreal Dexamethasone Implant for the Treatment of Uveitic Macular Edema: The PeriOcular vs. INTravitreal corticosteroids for uveitic macular edema (POINT) Trial. Ophthalmology. 2019; 126(2): 283-295.
17. Wang M, Hou LH, Wang F, et al. A clinical trial on Conbercept for macular edema secondary to central retinal vein occlusion. Int Eye Sci. 2017; 17(10): 1901-1904.
18. Yeh S, Khurana RN, Shah M, et al. Efficacy and Safety of Suprachoroidal CLS-TA for Macular Edema Secondary to Noninfectious Uveitis: Phase 3, Randomized Trial. Ophthalmology. 2020; 127(7): 948-955.
19. Zhang DW, Zhang R, Yang M, et al. Retrobulbar injection of triamcinolone acetonide combined with retinal photocoagulation in treatment of diabetic macular edema curative effect observation. Chinese Journal of Difficult and Complicated Cases. 2016; 15(12):1263-1267.
20. Yan J, Sun Z, Jiang X, et al. Clinical Evaluation on Triamcinolone Acetonide Combined with Modified Grid Laser Photocoagulation in the Treatment of Diabetic Macular Edema.China pharmaceuticals. 2019; 28(13): 56-58.
21. Yuan SM, Liu DN, Zhou XY. Vitreous cavity injection of ranibizumab vs its combination with triamcinolone acetonide in treatment of repeated or continuous CRVO-ME. Journal of the Third Military Medical University. 2015; 37(17): 1787-1791.
22. Alireza Ramezani, Hamed Esfandiari, Morteza Entezari, et al. Three intravitreal bevacizumab versus two intravitreal triamcinolone injections in recent onset central retinal vein occlusion.Acta Ophthalmol. 2014; 92: e530-e539.
23. David Leonardo Cruvinel Isaac, Murilo Batista Abud, Kariza Aiko Frantz, et al. Comparing intravitreal triamcinolone acetonide and bevacizumab injections for the treatment of diabetic macular oedema: a randomized doubleblind study. Acta Ophthalmol. 2012; 90: 56-60.
24. Sonoda S, Sakamoto T, Yamashita T, et al. Effect of intravitreal triamcinolone acetonide or bevacizumab on choroidal thickness in eyes with diabetic macular edema. Invest Ophthalmol Vis Sci. 2014; 55(6):3979-85.
25. Soheilian M, Garfami KH, Ramezani A, et al. Two-year results of a randomized trial of intravitreal bevacizumab alone or combined with triamcinolone versus laser in diabetic macular edema. Retina. 2012; 32(2):314-21.
26. N Shoeibi，H Ahmadieh，M Entezari，et al. Intravitreal Bevacizumab with or without Triamcinolone for Refractory Diabetic Macular Edema: Long-term Results of a Clinical Trial. J Ophthalmic Vis Res. 2013; 8 (2): 99-106.
27. Murilo W. Rodrigues, José A. Cardillo, André Messias, et al. Bevacizumab versus triamcinolone for persistent diabetic macular edema: a randomized clinical trial. Graefe's Archive for Clinical and Experimental Ophthalmology. 2020; 258: 479-490.
28. Riazi-Esfahani M, Riazi-Esfahani H, Ahmadraji A, et al. Intravitreal bevacizumab alone or combined with 1 mg triamcinolone in diabetic macular edema: a randomized clinical trial. Int Ophthalmol. 2018; 38(2):585-598.
29. Paccola L, Costa RA, Folgosa MS, et al. Intravitreal triamcinolone versus bevacizumab for treatment of refractory diabetic macular oedema (IBEME study). Br J Ophthalmol. 2008; 92(1):76-80.
30. Rakhee, Ambade, Ajay, Ambade, M, Sagdeo. Effect of combined Intravitreal Injections of Bevacizumab and Triamcinolone Acetonide vs intravitreal Bevacizumab in Diffuse Diabetic Macular Edema. IOSR Journal of Dental and Medical Sciences. 2014; 13: 01-06.
31. Marey HM, Ellakwa AF. Intravitreal bevacizumab alone or combined with triamcinolone acetonide as the primary treatment for diabetic macular edema. Clinical Ophthalmology (Auckland, N.Z.). 2011; 5: 1011-1016.
32. K Kriechbaum, S Prager, G Mylonas, et al. Intravitreal bevacizumab (Avastin) versus triamcinolone (Volon A) for treatment of diabetic macular edema: one-year results.Eye. 2014; 28: 9-16.
33. Ahmadieh, H., Ramezani, A., Shoeibi, N. et al. Intravitreal bevacizumab with or without triamcinolone for refractory diabetic macular edema; a placebo-controlled, randomized clinical trial. Graefes Arch Clin Exp Ophthalmol. 2008; 246: 483-489.

## Q9 on glucocorticoids for Vogt-Koyanagi-Harada (VKH) disease

**Recommendation 1**

**PICO framework**

Patient: patients with VKH disease

Intervention: intravenous pulse sequential oral glucocorticoid

Comparison: oral glucocorticoid

Outcome: all outcomes of interest were listed in the eTable below.

### Table S12. Summary of Findings and Certainty of Evidence Using GRADE-CERQual Approach

| **Outcomes** | **Study design** | **Methodological limitations** | **Coherence** | **Relevance** | **Adequacy** | **Summary of findings** | **CERQual rating** |
| --- | --- | --- | --- | --- | --- | --- | --- |
| Recurrence rate^1^ | 1 cohort study | Moderate | Not applicable | High | Low | The recurrence rate was significantly lower in the intravenous infusion plus oral glucocorticoid group than in the oral alone group | Very low |
| Visual acuity^1-3^ | 3 cohort studies | Low | Moderate | High | High, involving 3 countries | In terms of visual acuity improvement, different studies have shown inconsistent results. No significant differences were observed in either study. | Very low |
| Anterior chamber inflammation^3^ | 1 cohort study | Low | Not applicable | High | Low | After 6 months of treatment, no significant difference was observed. | Very low |
| Maximum detachment height of neuroepithelium^4^ | 1 RCT | Moderate | Not applicable | High | Low | There was a significant difference between the intravenous shock plus oral glucocorticoid group and the oral glucocorticoid group only after 5 days of treatment | Very low |
| Cataract^1,2^ | 2 cohort studies | Moderate | Low | High | Moderate, involving 2 countries | No significant differences were observed in either study. | Very low |
| Glaucoma^1^ | 1 cohort study | Moderate | Not applicable | High | Low | Glaucoma in the intervention group was significantly less than that in control group. | Very low |
| Systemic adverse events^4^ | 1 RCT | Moderate | Not applicable | High | Low | One patient in the intravenous shock plus oral group had mild gastrointestinal discomfort on the 4th day of treatment, and no gastrointestinal discomfort was reported in the oral group alone | Very low |

Abbreviations: RCT=randomized controlled trial.

**Recommendation 2**

**PICO framework**

Patient: patients with VKH disease

Intervention: systemic glucocorticoid combined with intraocular triamcinolone acetonide

Comparison: systemic glucocorticoid alone

Outcome: all outcomes of interest were listed in the eTable below.

### Table S13. Summary of Findings and Certainty of Evidence Using GRADE-CERQual Approach

| **Outcomes** | **Study design** | **Methodological limitations** | **Coherence** | **Relevance** | **Adequacy** | **Summary of findings** | **CERQual rating** |
| --- | --- | --- | --- | --- | --- | --- | --- |
| Visual acuity^5-7^ | 3 RCTs | Low | Moderate | High | Low, involving 1 country | The results of three RCT studies were inconsistent. | Very low |
| Neural cortical thickness^5,7^ | 2 RCTs | Low | High | High | Low, involving 1 country | After 1 week, 1 month, 3 months and 8 months of treatment, the thickness of the macular retinal nerve cortex in the triamcinolone acetonide group was significantly lower than that in the systemic glucocorticoid group. | Very low |
| Retinal fluorescence leakage disappeared^5,6^ | 2 RCTs | Low | Low | High | Low, involving 1 country | Retinal vascular fluorescence leakage was improved to varying degrees in most patients after treatment in the topical triamcinolone acetonide group. More eyes in the triamcinolone acetonide group recovered from leakage. | Very low |
| Cataract^5^ | 1 RCT | Low | Not applicable | High | Low | No secondary cataract was found in both groups | Very low |
| Glaucoma and elevated IOP^5^ | 1 RCT | Low | Not applicable | High | Low | Glaucoma was not found in both groups, and there was no significant difference in IOP between the two groups after 1 month of treatment | Very low |

Abbreviations: IOP= intraocular pressure; RCT=randomized controlled trial.

**Drug approval status in the United States, the European Union, and China**

None.

**Reference**

1. Park UC, Cho IH, Lee EK, Yu HG. The effect on choroidal changes of the route of systemic corticosteroids in acute vogt-koyanagi-harada disease. Graefe's archive for clinical and experimental ophthalmology = Albrecht von Graefes Archiv fur klinische und experimentelle Ophthalmologie. 2017;255(6):1203-1211.
2. Read RW, Yu F, Accorinti M, Bodaghi B, Chee SP, Fardeau C, Goto H, Holland GN, Kawashima H, Kojima E, Lehoang P, Lemaitre C, Okada AA, Pivetti-Pezzi P, Secchi A, See RF, Tabbara KF, Usui M, Rao NA. Evaluation of the effect on outcomes of the route of administration of corticosteroids in acute vogt-koyanagi-harada disease. American journal of ophthalmology. 2006;142(1):119-124.
3. Sasamoto Y, Ohno S, Matsuda H. Studies on corticosteroid therapy in vogt-koyanagi-harada disease. Ophthalmologica Journal international d'ophtalmologie International journal of ophthalmology Zeitschrift fur Augenheilkunde. 1990;201(3):162-167.
4. Wang LB, Yu DY, Zhou X. [Optical coherence tomography analysis the effect of methylprednisolone on Vogt-Koyanagi-Harada syndrome]. Anhui medical and pharmaceutical journal. 2017;21(11):2076-2080.
5. Wang B, Gao F, Liu X, Zhao Q. [Clinical efficacy and perioperative nursing care of intravitreal injected triamcinolone acetonide for Vogt-Koyanagi-Harada disease]. Chinese journal of modern nursing. 2015;21(13):1533-1536.
6. Yang SL, Guo Y, Sun YJ. [Clinical observation of Vogt-Koyanagi-Harada (VKH) disease by injecting triamcinolone acetonide (TA) under Tenon’s]. Journal of hebei medical university. 2013;34(6):711-713.
7. Yang SL, Guo Y. [Clinic effect on macular serous retinal detachment of Vogt-Koyanagi-Harada (VKH) disease by injecting triamcinolone acetonide (TA) under Tenon’s]. Chinese journal of practical ophthalmology. 2013;31(5):575-577.

## Q10 on cyclosporine for VKH disease

**Recommendation 1**

**PICO framework**

Patient: patients with VKH disease

Intervention: cyclosporine therapy plus oral glucocorticoid

Comparison: glucocorticoid

Outcome: all outcomes of interest were listed in the eTable below.

### Table S14. Summary of Findings and Certainty of Evidence Using GRADE-CERQual Approach

| **Outcomes** | **Study design** | **Methodological limitations** | **Coherence** | **Relevance** | **Adequacy** | **Summary of findings** | **CERQual rating** |
| --- | --- | --- | --- | --- | --- | --- | --- |
| **Oral glucocorticoid plus cyclosporine vs. intravenous pulse plus oral glucocorticoid** | | | | | | | |
| Recurrence rate^1^ | 1 RCT | Moderate | Not applicable | High | Low | No significant difference in recurrence rates was observed between the intervention and control groups. | Very low |
| Visual acuity^1,2^ | 2 RCTs | Low | Low | High | Moderate, involving 2 countries | Results varied between studies. One study (n=36) found that cyclosporine therapy plus oral glucocorticoid gained better visual acuity. | Low |
| Fundus changes^1,2^ | 2 RCTs | Low | Moderate | High | Moderate, involving 2 countries | Basically, the same results were obtained between studies. The cyclosporine group was better in improving fundus vascular fluorescence leakage at 1 month, and the sunset fundus index from 6 to 12 months was significantly better than the non-cyclosporine group. | Low |
| Cataract^1^ | 1 RCT | Moderate | Not applicable | High | Low | The cataract of the intravenous pulse plus oral glucocorticoid group was significantly higher than that of oral glucocorticoid plus cyclosporine at 6 and 12 months. | Very low |
| Drug withdrawal due to adverse events^1^ | 1 RCT | Moderate | Not applicable | High | Low | Three patients in the oral glucocorticoid plus cyclosporine group were discontinued due to adverse events. | Very low |
| **Oral glucocorticoids plus cyclosporine vs. oral glucocorticoids** | | | | | | | |
| Recurrence^3^ | 1 cohort study | Low | Not applicable | Moderate | Low | A patient discontinued cyclosporine due to renal impairment and relapsed 4 months after treatment | Very low |
| Visual acuity^4^ | 1 RCT | Low | Not applicable | Moderate | Low | After treatment, the visual acuity of cyclosporine group was significantly better than that of control group. | Very low |
| Glaucoma, cataract^4^ | 1 RCT | Low | Not applicable | Moderate | Low | There was no significant difference between the two groups | Very low |
| Systemic adverse events^3,4^ | 1 RCT  1 cohort study | Low | High | Moderate | Low | There was no significant difference between the two groups | Very low |

Abbreviations: RCT=randomized controlled trial.

**Recommendation 2**

**PICO framework**

Patient: patients with VKH disease who did not respond well to glucocorticoid

Intervention: glucocorticoid plus cyclosporine

Comparison: glucocorticoid plus azathioprine

Outcome: all outcomes of interest were listed in the eTable below.

### Table S15. Summary of Findings and Certainty of Evidence Using GRADE-CERQual Approach

| **Outcomes** | **Study design** | **Methodological limitations** | **Coherence** | **Relevance** | **Adequacy** | **Summary of findings** | **CERQual rating** |
| --- | --- | --- | --- | --- | --- | --- | --- |
| Recurrence^5^ | 1 RCT | Moderate | Not applicable | Moderate | Low | Two of the 9 patients in the cyclosporine group relapsed after discontinuing cyclosporine, and 6 of the 12 patients in the azathioprine group relapsed after 6-12 months of treatment | Low |
| Visual acuity^5^ | 1 RCT | Moderate | Not applicable | Moderate | Low | There was no significant difference between the cyclosporine group and azathioprine group in visual acuity improvement after treatment | Low |
| Anterior chamber inflammation^5^ | 1 RCT | Moderate | Not applicable | Moderate | Low | There was no significant difference between cyclosporine group and azathioprine group | Low |
| Reduced glucocorticoid use^5^ | 1 RCT | Moderate | Not applicable | Moderate | Low | At 6 months, glucocorticoid use in the cyclosporine group was significantly lower than that in the azathioprine group, but at 12 months, the cumulative glucocorticoid dose in the two groups was not significant | Low |
| Drug withdrawal due to adverse events^5^ | 1 RCT | Moderate | Not applicable | Moderate | Low | Three patients in the azathioprine group stopped taking the drug due to herpes zoster, while the cyclosporine group did not stop taking the drug due to adverse events, showing no significant difference between the two groups | Low |
| Ocular adverse events^5^ | 1 RCT | Moderate | Not applicable | Moderate | Low | There were no significant differences in cataract, subretinal fibrosis, depigmentation, ectopic retinal pigment epithelium, glaucoma and optic disc edema | Low |
| Non-ocular adverse events^5^ | 1 RCT | Moderate | Not applicable | Moderate | Low | Non-ocular adverse events were reported in 8 patients in the azathioprine group and 6 patients in the cyclosporine group, with no significant difference between the two groups | Low |

Abbreviations: RCT=randomized controlled trial.

**Drug approval status in the United States, the European Union, and China**

**In the United States and the European Union**, cyclosporine was not approved for VKH disease.

**In China**, cyclosporine was approved for endogenous uveitis.

**Reference**

1. Ono T, Mochizuki M, Goto H, Sakai T, Nitta F, Mizuki N, Takase H, Kaneko Y, Hori J, Nakano S, Nao-I N, Ohguro N. A prospective multi-center randomized clinical study comparing steroid-pulse therapy and a combined therapy with oral prednisolone and cyclosporine for new-onset acute vogt-koyanagi-harada disease. Investigative Ophthalmology and Visual Science. 2020;61(7). Meeting abstract.
2. Liu H, Zhang WC, Gu WZ. [Clinical Study of CSA Combined with Glucocorticoid in the Treatment of Vogt-Koyanagi-Harada Disease]. Aerospase medicine. 2010;21(06):871-873.
3. Chen XM, Ou JX, Zhang XF, Liao RD, Ma CP. [Clinical characteristics and treatment of Vogt-koyanagi-harada syndrome]. Guangdong medical journal. 2004;25(7):836-838.
4. Wang NN. [Clinical analysis of cyclosporine A combined with glucocorticoid in the treatment of recurrent vogt- Koyanagi Harada disease]. Chinese journal of modern drug application. 2016;10(16):157-158.
5. Cuchacovich M, Solanes F, Díaz G, Cermenati T, Avila S, Verdaguer J, Verdaguer JI, Carpentier C, Stopel J, Rojas B, Traipe L, Gallardo P, Sabugo F, Zanoli M, Merino G, Villarroel F. Comparison of the clinical efficacy of two different immunosuppressive regimens in patients with chronic vogt-koyanagi-harada disease. Ocular immunology and inflammation. 2010;18(3):200-207.

## Q11 on prednisolone for Behcet's disease

**Recommendation**

**PICO framework**

Patient: patients with Behcet's disease

Intervention: prednisolone, methylprednisolone

Comparison: other treatments

Outcome: all outcomes of interest were listed in the eTable below.

### Table S16. Summary of Findings and Certainty of Evidence Using GRADE-CERQual Approach

| **Outcomes** | **Study design** | **Methodological limitations** | **Coherence** | **Relevance** | **Adequacy** | **Summary of findings** | **CERQual rating** |
| --- | --- | --- | --- | --- | --- | --- | --- |
| Visual acuity^1-5^ | 1 RCT  4 case series | Moderate | Moderate | Low | Low, involving 3 countries | 4 articles had improved visual acuity, and one had poor prognosis. | Low |
| Disease Activity Index (DAI)^1,2^ | 1 RCT  1 case series | Moderate | Low | Moderate | Low, involving 2 countries | There was no significant difference in DAI in 1 study, and DAI in 1 study was improved. | Low |
| Total Inflammatory Activity Index (TIAI)^1,3^ | 1 RCT  1 case series | Moderate | High | Moderate | Low, involving 2 countries | TIAI in both studies were improved. | Moderate |
| Adverse events^1,4^ | 1 RCT  1 case series | Moderate | High | Moderate | Low, involving 2 countries | One RCT indicated that there was no statistically significant difference between intravenous and oral glucocorticoid versus oral glucocorticoid, and almost all patients showed signs of glucocorticoid overdose, such as weight gain or one-month face. | Moderate |

Abbreviations: RCT=randomized controlled trial.

**Drug approval status in the United States, the European Union, and China**

None.

**Reference**

1. Mohammadi M, Shahram F, Shams H, Akhlaghi M, Ashofteh F, et al. High-dose intravenous steroid pulse therapy in ocular involvement of Behcet's disease: a pilot double-blind controlled study. Int J Rheum Dis. 2017; 20(9):1269-1276.
2. Dacatchi F, Shams H, Shahram F, Nadji A, Chams-Davatchi C, et al. Methotrexate in ocular manifestations of Behcet's disease: a longitudinal study up to 15 years.Int J Rheum Dis. 2013; 16(5):568-577.
3. Benezra D, Cohen E. Treatment and visual prognosis in Behçet's disease. Br J Ophthalmol. 1986;70(8):589-592.
4. Zhao M, Wang H, Jiao X, Wei WB. Clinical observation of 85 patients with Behcet's uveitis treated with immunosuppressive agents combined with steroids. Clin J Ophthalmol. 2013; 49(3): 202-206.
5. Zhang MF, Zhao C, Wen X, Du H, Zhao Y. The short-term efficacy and safety treatment study of recurrent nveitis in Behcet's disease with etanercep. Clin J Ophthalmol. 2010; 46(2): 145-150.

## Q12 on systemic glucocorticoids for optic neuritis

**Recommendation**

**PICO framework**

Patient: patients with optic neuritis

Intervention: oral or intravenous glucocorticoid

Comparison: placebo

Outcome: all outcomes of interest were listed in the eTable below

### Table S17. Summary of Findings and Certainty of Evidence Using GRADE Approach^1^

| **Quality assessment** | | | | | | | | **Summary of findings** | | | |
| --- | --- | --- | --- | --- | --- | --- | --- | --- | --- | --- | --- |
|  |  |  |  |  |  |  |  | **Number of patients** | | **Effect size (95%CI)** | **Certainty of evidence** |
| **Outcomes** | **Number of included studies** | **Study design** | **Risk of bias** | **Inconsistency** | **Indirectness** | **Imprecision** | **Publication bias** | **Intervention group** | **Control group** |  |  |
| **Oral glucocorticoid vs. placebo** | | | | | | | | | | | |
| Visual acuity at 1 month | 3 | RCT | General | None | None | Serious | None | 96/194 | 96/204 | RR = 1.00 (0.82, 1.23) | Moderate |
| Visual acuity at 6 months | 2 | RCT | General | None | None | Serious | None | 94/171 | 111/184 | RR = 0.92 (0.77, 1.11) | Moderate |
| Visual acuity at 1 year | 3 | RCT | General | General | None | Serious | None | 102/176 | 136/192 | RR = 0.93 (0.7, 1.24) | Moderate |
| Contrast sensitivity at 1 month | 2 | RCT | General | None | None | Serious | None | 126/178 | 119/170 | RR = 1.00 (0.90, 1.12) | Moderate |
| Contrast sensitivity at 1 year | 2 | RCT | General | None | None | Serious | None | 130/162 | 131/159 | RR = 1.04 (0.64, 1.69) | Moderate |
| vision at 1 month | 2 | RCT | General | None | None | Serious | None | 78/162 | 71/174 | RR = 1.23 (0.97, 1.56) | Moderate |
| June vision at 6 months | 2 | RCT | General | None | None | Serious | None | 129/170 | 142/184 | RR = 1.01 (0.91, 1.13) | Moderate |
| Visual acuity at 1 year | 2 | RCT | General | None | None | Serious | None | 102/154 | 121/166 | RR = 0.96 (0.85, 1.09) | Moderate |
| **Intravenous glucocorticoid vs. placebo** | | | | | | | | | | | |
| Visual acuity at 6 months | 2 | RCT | General | None | None | Serious | None | 102/173 | 96/173 | RR = 1.05 (0.88, 1.26) | Moderate |
| Contrast sensitivity at 1 month | 2 | RCT | General | General | None | None | None | 131/158 | 122/164 | RR = 1.27 (0.74, 2.16) | High |
| Contrast sensitivity at 6 months | 2 | RCT | General | None | None | Serious | None | 105/173 | 95/173 | RR = 1.11 (0.92, 1.33) | Moderate |
| Visual acuity at 1 month | 2 | RCT | General | None | None | None | None | 87/168 | 59/162 | RR = 1.56 (0.88, 2.76) | High |
| Visual acuity at 6 months | 2 | RCT | General | None | None | Serious | None | 134/173 | 124/173 | RR = 1.08 (0.96, 1.21) | Moderate |
| Visual acuity at 1 year | 2 | RCT | General | None | None | Serious | None | 104/162 | 98/154 | RR 1.01 (0.86, 1.19) | Moderate |

Abbreviations: RCT=randomized controlled trial; RR=relative risk.

**Drug approval status in the United States, the European Union, and China**

**In the United States and the European Union**, none.

**In China**, methylprednisolone was approved for optic neuritis.

**Reference**

1. Gal RL, Vedula SS, Beck R. Corticosteroids for treating optic neuritis. Cochrane Database Syst Rev. 2015(8):CD001430.

## Q13 on acyclovir for acute retinal necrosis

**Recommendation**

**PICO framework**

Patient: patients with acute retinal necrosis

Intervention: acyclovir

Comparison: non-acyclovir treatment

Outcome: all outcomes of interest were listed in the eTable below.

### Table S18. Summary of Findings and Certainty of Evidence Using GRADE-CERQual Approach

| **Outcomes** | **Study design** | **Methodological limitations** | **Coherence** | **Relevance** | **Adequacy** | **Summary of findings** | **CERQual rating** |
| --- | --- | --- | --- | --- | --- | --- | --- |
| The contralateral eye involved in monocular disease^1,2^ | 1 cohort study  1 case series | Moderate | Moderate | High | Moderate | Acyclovir treatment reduced contralateral involvement in patients with monocular disease compared with non-acyclovir treatment | Moderate |
| Visual acuity^2-6^ | 5 case series | Low | High | Moderate | Low | In four of the five studies, acyclovir was used in combination with laser and surgery to improve visual acuity. | Very low |

**Drug approval status in the United States, the European Union, and China**

None.

**Reference**

1. Palay DA, Sternberg PJ, Davis J, et al. Decrease in the risk of bilateral acute retinal necrosis by acyclovir therapy. American journal of ophthalmology. 1991;112(3):250-255.
2. Dong WH, Zhou F, Bi HS, et al. [Clinical treatment of 42 eyes with acute retinal necrosis syndrome]. Journal of Otolaryngology and Ophthalmology of Shandong University. 2008(05):457-459+463.
3. Liu J, Dou HG. [Treatment of acute retinal necrosis syndrome]. International Journal of Ophthalmology. 2008(02):416-418.
4. You CT, Jin XM, Zhang XF. [Clinical treatment of acute retinal necrosis syndrome]. Chinese Journal of Ophthalmology in Ophthalmic Trauma. 2012; (08):588-591.
5. Wang ZG. [To explore the clinical treatment effect of acute retinal necrosis syndrome]. Electronic Journal of Clinical Medicine Literature. 2017; 4(31):5995.
6. Roy R, Pal BP, Mathur G, Rao C, Das D, Biswas J. Acute retinal necrosis: clinical features, management and outcomes, a 10-year consecutive case series. Ocular immunology and inflammation. 2014;22(3):170-174.

## Q14 on intraoperative and postoperative use of mitomycin C (MMC) for glaucoma

**Recommendation 1**

**PICO framework**

Patient: glaucoma patients undergoing trabeculectomy

Intervention: mitomycin used intraoperatively

Comparison: placebo

Outcome: all outcomes of interest were listed in the eTable below.

### Table S19. Summary of Findings and Certainty of Evidence Using GRADE Approach

| **Quality assessment** | | | | | | | | **Summary of findings** | | | |
| --- | --- | --- | --- | --- | --- | --- | --- | --- | --- | --- | --- |
|  |  |  |  |  |  |  |  | **Number of patients** | | **Effect size (95%CI)** | **Certainty of evidence** |
| **Outcomes** | **Number of included studies** | **Study design** | **Risk of bias** | **Inconsistency** | **Indirectness** | **Imprecision** | **Publication bias** | **Intervention group** | **Control group** |  |  |
| IOP^2-15^ | 13 | RCT | Serious | Serious | None | General | None | 350 | 328 | MD = -10.99 mmHg (-18.52, -3.47) | Low |
| Filtering bleb^10-16^ | 7 | RCT | Serious | None | None | General | None | 377 | 359 | RR= 1.18 (1.09, 1.27) | Moderate |
| Surgical failure rate^2-9,17-19^ | 11 | RCT | Serious | None | None | General | None | 423 | 275 | RR = 0.43 (0.28, 0.66) | Moderate |
| Low IOP^2-7,9,14,16-20^ | 13 | RCT | Serious | None | None | General | None | 408 | 353 | RR = 0.03 (0.03, 3.06) | Moderate |
| Anterior chamber bleeding^2-9,12,13,15,17,19^ | 13 | RCT | General | None | None | General | None | 612 | 379 | RR = 0.93 (0.41, 2.08) | Moderate |
| Filtering bleb leakage^3-5,7-9,12,15,19^ | 9 | RCT | Serious | None | None | General | None | 275 | 215 | RR = 1.25 (0.63, 2.47) | Moderate |
| Hypohypertensive macular degeneration^11,15,16^ | 3 | RCT | Serious | None | None | General | None | 275 | 215 | RR = 1.91 (0.50, 7.25) | Moderate |
| Endophthalmitis^3,7,17^ | 3 | RCT | Serious | None | None | General | None | 71 | 36 | RR = 1.96 (0.23, 16.89) | Moderate |
| Shallow anterior chamber^2-10,12,15-17,19,20^ | 15 | RCT | Serious | None | None | General | None | 71 | 36 | RR = 1.65 (1.05, 2.62) | Moderate |

Abbreviations: IOP= intraocular pressure; RCT=randomized controlled trial; RR=relative risk.

**Recommendation 2**

**PICO framework**

Patient: glaucoma patients who underwent bleb needling after trabeculectomy failure

Intervention: mitomycin used intraoperatively

Comparison: placebo

Outcome: all outcomes of interest were listed in the eTable below

### Table S20. Summary of Findings and Certainty of Evidence Using GRADE Approach^21^

| **Quality assessment** | | | | | | | | **Summary of findings** | | | |
| --- | --- | --- | --- | --- | --- | --- | --- | --- | --- | --- | --- |
|  |  |  |  |  |  |  |  | **Number of patients** | | **Effect size (95%CI)** | **Certainty of evidence** |
| **Outcomes** | **Number of included studies** | **Study design** | **Risk of bias** | **Inconsistency** | **Indirectness** | **Imprecision** | **Publication bias** | **Intervention group** | **Control group** |  |  |
| **MMC vs. no anti-metabolite** | | | | | | | | | | | |
| IOP | 2 | comparative studies | General | None | None | Serious | None | 29 | 19 | MD=-1.67 mmHg (-4.35, -1.00) | Very low |
| Surgical success | 2 | comparative studies | General | None | None | Serious | None | 29 | 19 | RR=1.32 (0.72, 2.39) | Very low |
| Hypotony | 1 | comparative studies | General | None | None | Serious | None | 16 | 16 | RR=0.50 (95% CI 0.05 to 5.04) | Very low |
| **MMC vs. 5-Fu** | | | | | | | | | | | |
| IOP | 4 | 1 RCT plus 3 comparative studies | General | None | None | General | None | 136 | 160 | MD=0.20 (-1.00 to 1.39) mmHg | Low |
| Surgical success | 3 | 1 RCT plus 2 comparative studies | General | None | None | General | None | 94 | 63 | RR=0.93 (0.46, 1.88) | Low |
| Hypotony | 2 | comparative studies | General | None | None | General | None | 81 | 85 | RR=0.68 (0.12, 3.74) | Low |

Abbreviations: IOP= intraocular pressure; RCT=randomized controlled trial; MD=mean difference; RR=relative risk.

**PICO framework**

Patient: glaucoma patients who underwent bleb needling after trabeculectomy failure

Intervention: mitomycin used intraoperatively

Outcome: all outcomes of interest from baseline to the last visit were listed in the eTable below.

### Table S21. Summary of Findings and Certainty of Evidence Using GRADE-CERQual Approach^22^

| **Outcomes** | **Study design** | **Methodological limitations** | **Coherence** | **Relevance** | **Adequacy** | **Summary of findings** | **CERQual rating** |
| --- | --- | --- | --- | --- | --- | --- | --- |
| IOP | 10 case series plus 4 comparative studies | Moderate | High | High | Moderate | The overall effects of bleb needling with MMC revealed a reduction in IOP of MD=-9.72 mmHg (95%CI -8.41 to -11.03) from baseline to the last visit. | Moderate |
| Postoperative number of anti-glaucoma medications | 10 case series plus 2 comparative studies | Moderate | High | Moderate | Moderate | Postoperative number of anti-glaucoma medications was reduced than the preoperative number of medications by MD=0.80 (0.73, 1.53). | Moderate |

Abbreviations: IOP= intraocular pressure; MD=mean difference.

**Drug approval status in the United States, the European Union, and China**

None.

**Reference**

1. Wilkins M, Indar A, Wormald R. Intra-operative mitomycin C for glaucoma surgery. Cochrane Database Syst Rev. 2005(4):CD002897.
2. Andreanos D, Georgopoulos GT, Vergados J, Papaconstantinou D, Liokis N, Theodossiadis P. Clinical evaluation of the effect of mitomycin-C in re-operation for primary open angle glaucoma. European journal of ophthalmology. 1997;7(1):49-54.
3. Carlson DW, Alward WL, Barad JP, Zimmerman MB, Carney BL. A randomized study of mitomycin augmentation in combined phacoemulsification and trabeculectomy. Ophthalmology. 1997;104(4):719-724.
4. Cohen JS, Greff LJ, Novack GD, Wind BE. A placebo-controlled, double-masked evaluation of mitomycin C in combined glaucoma and cataract procedures. Ophthalmology. 1996;103(11):1934-1942.
5. Costa VP, Comegno PE, Vasconcelos JP, Malta RF, José NK. Low-dose mitomycin C trabeculectomy in patients with advanced glaucoma. Journal of glaucoma. 1996;5(3):193-199.
6. Martini E, Laffi GL, Sprovieri C, Scorolli L. Low-dosage mitomycin C as an adjunct to trabeculectomy. A prospective controlled study. European journal of ophthalmology. 1997;7(1):40-48.
7. Shin DH, Hughes BA, Song MS, et al. Primary glaucoma triple procedure with or without adjunctive mitomycin. Prognostic factors for filtration failure. Ophthalmology. 1996;103(11):1925-1933.
8. Turaçli E, Gündüz K, Aktan G, Tamer C. A comparative clinical trial of mitomycin C and cyclosporin A in trabeculectomy. European journal of ophthalmology. 1996;6(4):398-401.
9. Wu L, Yin J. [The effect of mitomycin C on filtration surgery of glaucoma with poor prognosis]. Chinese Journal of Ophthalmology 1996;32(1):32–4.
10. Huang X. [A clinical study of compound trabeculectomy in the treatment of glaucoma patients]. China Medical Herald. 2012;9(04):28-29.
11. Zheng X, Ma WN, Ye CH. [The effect of 3 mitomycin C on corneal endothelial cells in patients with cataract and glaucoma]. International Journal of Ophthalmology. 2018;18(01):96-99.
12. Li Q, Liu W. [Intraoperative application of 5 mitomycin C in the treatment of 98 cases of refractory glaucoma]. Chinese Journal of Difficult and Complicated Cases. 2012, 11(10):769-771.
13. Liu S, Xing SH. [Clinical efficacy of 6 trabeculectomy combined with mitomycin C in the treatment of glaucoma]. International Journal of Ophthalmology. 2014;14(12):2256-2258.
14. Zhao RZ, Zhao FZ. [Efficacy observation of trabeculectomy combined with mitomycin C in the treatment of glaucoma]. Shandong Medicine. 2012;52(21):87-88.
15. Liu YD, Li J, Chen HJ, Zhao Y, Li D. [Observation on the efficacy of 8 trabeculectomy combined with mitomycin C in the treatment of glaucoma]. Shandong Medicine. 2010;50(02):105-106.
16. Quan G, Hong S. [Influence of mitomycin C to intraocular pressure and blood flow of glaucoma patients after trabeculectomy]. International Eye Science. 2015, (7):1220-1222.
17. Robin AL, Ramahrishnan R, Krishnadas R, et al. A long-term dose-response study of mitomycin in glaucoma filtration surgery. Archives of Ophthalmology. 1997;115(8):969-974.
18. Shin DH, Kim YY, Sheth N, et al. 16-The role of adjunctive mitomycin C in secondary glaucoma triple procedure as compared to primary glaucoma triple procedure. Ophthalmology. 1998;105(4):740-745.
19. Szymaǹski A, Gierek-Łapiǹska A, Koziak M, Gierek-Ciaciura S. 17A fluorophotometric study of corneal endothelium after trabeculectomy using different concentrations of Mitomycin-C. International ophthalmology. 1996;20(1-3):95-99.
20. Li J, Bo WX. [Clinical efficacy and safety of glaucoma filtration surgery combined with the application of 5-fluorouracil]. International Eye Science. 2015, (1):38-40
21. Chen X, Suo L, Hong Y, Zhang C. Safety and Efficacy of Bleb Needling with Antimetabolite after Trabeculectomy Failure in Glaucoma Patients: A Systemic Review and Meta-Analysis. Journal of ophthalmology. 2020:4310258.
22. Halili A, Kessel L, Subhi Y, Bach-Holm D. Needling after trabeculectomy - does augmentation by anti-metabolites provide better outcomes and is Mitomycin C better than 5-Fluoruracil? A systematic review with network meta-analyses. Acta ophthalmologica. 2020;98(7):643-653.

## Q15 on isosorbide for glaucoma

**Recommendation**

**PICO framework**

Patient: patients with glaucoma

Intervention: oral isosorbide

Comparison: placebo or other osmotic agents

Outcome: all outcomes of interest were listed in the eTable below.

### Table S22. Summary of Findings and Certainty of Evidence Using GRADE-CERQual Approach

| **Outcomes** | **Study design** | **Methodological limitations** | **Coherence** | **Relevance** | **Adequacy** | **Summary of findings** | **CERQual rating** |
| --- | --- | --- | --- | --- | --- | --- | --- |
| Intraocular pressure^1-5^ | 5 case series | Moderate | High | High | Low, involving 2 countries | A single oral administration of isosorbide solution 1-2mg/kg reduced intraocular pressure in glaucoma patients. | Very Low |

**Drug approval status in the United States, the European Union, and China**

None.

**Reference**

1. Wisznia KI, Lazar M, Leopold IH. Oral isosorbide and intraocular pressure. Am J Ophthalmol. 1970;70(4):630-634.

2. Mehra KS, Singh R, Char JN, Rajyashree K. Lowering of intraocular tension. Effects of isosorbide and glycerin. Arch Ophthalmol. 1971;85(2):167-168.

3. Kulshrestha OP, Mittal RN. Isosorbide and intraocular pressure. Br J Ophthalmol. 1972;56(5):439-441.

4. Shen ZM, Yuan SY, Wang ZY. [Clinical observation of oral administration of isosorbide to lower intraocular pressure]. New Medicine. 1977; (Z2):507-508.

5. Wang XH, Yang LL. [Clinical study of isosorbide oral solution for lowering intraocular pressure]. Chinese Journal of Clinical Medicine (Guangzhou). 2003; 66: 50-51.

## Q16 on atropine in the prophylactic treatment of malignant (ciliary block) glaucoma

**Recommendation**

**PICO framework**

Patient: patients with malignant (ciliary block) glaucoma

Intervention: atropine eye drops combined with other drug treatment

Outcome: all outcomes of interest were listed in the eTable below.

### Table S23. Summary of Findings and Certainty of Evidence Using GRADE-CERQual Approach

| **Outcomes** | **Study design** | **Methodological limitations** | **Coherence** | **Relevance** | **Adequacy** | **Summary of findings** | **CERQual rating** |
| --- | --- | --- | --- | --- | --- | --- | --- |
| Response rate^1-13^ | 13 case series | Moderate | High | High | Low, involving 2 countries | A total of 351 eyes of 315 patients were included, and 63 eyes of 58 patients were relieved after combined drug treatment with 1% atropine eye drops, avoiding surgical treatment, with a total remission rate of 17.95%, ranging from 5.56% to 61.54%. No adverse events were reported, and no aggravation was found. | Very Low |

**Drug approval status in the United States, the European Union, and China**

None.

**Reference**

1. Yang HY, Xuan J, Wei QY. [Discussion on the treatment of ciliary ring obstructive glaucoma]. Journal of Ophthalmology of Ophthalmic Trauma. 2003; 25(5):1.

2. Zeng LZ, Zhang YT. [Clinical analysis of non-surgical ciliary ring block glaucoma]. Chinese Journal of Practical Ophthalmology. 2005; 23(8):2.

3. He YQ. [Discussion on the treatment of ciliary ring obstructive glaucoma]. Chinese Journal of Ophthalmic Trauma Occupational Ophthalmology. 2005; 27(007):521-522.

4. Tian F, Zhang Hong, Li XR, et al. [Combined treatment of ciliary ring obstructive glaucoma]. Journal of Ophthalmology of Ophthalmic Trauma. 2006; 28(9):3.

5. Fan PS, Li ZM, Chen XK. [Experience in clinical treatment of malignant glaucoma]. Clinical Medicine Practice. 2012; 21(2):2.

6. Zhang WZ, Huang L, Ma J, et al. [Clinical analysis of malignant glaucoma]. Chinese Journal of Ophthalmology. 2013; 49(2):4.

7. Dave P, Senthil S, Rao HL, Garudadri CS. Treatment outcomes in malignant glaucoma. Ophthalmology. 2013;120(5):984-990.

8. Wang YR, Kong XL, Jin ZL, et al. [Clinical analysis of stepwise treatment of ciliary ring block glaucoma]. New Advances in Ophthalmology. 2014; 34(11):4.

9. Wang M, Tan Q, Jiang HB, et al. [Clinical analysis of malignant glaucoma after glaucoma surgery]. Journal of Central South University: Medical Edition. 2015; (5):6.

10. Wang XZ, Wang XT, Wu HC, et al. [Experience in clinical treatment of ciliary ring obstructive glaucoma]. Practical Blind Prevention Technology. 2016;(4):3.

11. Wu ZH, Wang YH, Liu Y. Management strategies in malignant glaucoma secondary to antiglaucoma surgery. Int J Ophthalmol. 2016;9(1):63-68. Published 2016 Jan 18.

12. Liang L, Liu Q, Ma JJ, et al. [Clinical analysis of malignant glaucoma after phacoemulsification]. International Journal of Ophthalmology. 2019; 19(5):3.

13. Chen XL, Bin L, Xu ZK. [Efficacy analysis of personalized regimen in the treatment of malignant glaucoma]. International Journal of Ophthalmology. 2020; 20(2):3.

## Q17 on tacrolimus eye drops for corneal transplantation

**Recommendation**

**PICO framework**

Patient: corneal transplant patients

Intervention: tacrolimus eye drops

Comparison: other immunosuppressants

Outcome: all outcomes of interest were listed in the eTable below.

### Table S24. Summary of Findings and Certainty of Evidence Using GRADE Approach

| **Quality assessment** | | | | | | | | **Summary of findings** | | | |
| --- | --- | --- | --- | --- | --- | --- | --- | --- | --- | --- | --- |
|  |  |  |  |  |  |  |  | **Number of patients** | | **Effect size (95%CI)** | **Certainty of evidence** |
| **Outcomes** | **Number of included studies** | **Study**  **design** | **bias**  **risk** | **different**  **consistency** | **Indirectness** | **Imprecision** | **Publication bias** | **Intervention group** | **Control group** |  |  |
| Graft rejection^1-6^ | 6 | RCT | General | General | None | None | General | 47/185 | 81/185 | RR = 0.59 (0.38, 0.92) | High |
| Postoperative vision^2,5,6^ | 3 | RCT | General | None | None | None | General | 44/84 | 23/83 | RR =1.78 (1.13, 2.80) | High |
| Graft survival^1,5^ | 2 | RCT | General | Serious | None | Serious | General | 73/81 | 64/82 | RR = 1.12 (0.83, 1.52) | Low |
| Irreversible graft rejection^1,3,4^ | 2 | RCT | General | None | None | Serious | General | 3/56 | 6/56 | RR = 0.55 (0.08, 3.64) | Moderate |
| Ocular adverse events^2,5,6^ | 3 | RCT | General | Serious | None | None | General | 8/109 | 71/109 | RR = 0.06 (0.00, 1.42) | Moderate |

Abbreviations: RCT=randomized controlled trial; RR=relative risk.

**Drug approval status in the United States, the European Union, and China**

None.

**Reference**

1. Faramarzi, A, Abbasi, H, Feizi, , Hadi, Y, Azari, AA, Karimian, F. Topical 0.03% tacrolimus versus systemic mycophenolate mofetil as adjuncts to systemic corticosteroids for preventing graft rejection after repeat keratoplasty: one-year results of a randomized clinical trial. Eye (Lond), 2021. 35(10): 2879-2888.
2. Ye CT, Lin YS, Tang XL, Diao HX. Effects of tacrolimus eye drops and cyclosporine A eye drops on immune rejection after total keratoplasty. Today Pharmacy, 2004. 14(006): 43-44.
3. Zhai LY, Zhang XR, Liu H, Ma Y, Xu HC. Observation of topical tacrolimus on high-risk penetrating keratoplasty patients: a randomized clinical trial study. Eye (Lond), 2020. 34(9): 1600-1607.
4. Reinhard, T., Mayweg, S., Reis, A., Sundmacher, R. Topical FK506 as immunoprophylaxis after allogeneic penetrating normal-risk keratoplasty: a randomized clinical pilot study. Transpl Int, 2005. 18(2): 193-7.
5. Liao XM, Feng X, Shangguan GH. Effect of tacrolimus eye drops on immune rejection after high risk corneal transplantation. Chinese Pharmacists, 2020(3): 4.
6. Wang MH, Lin YSh, Chen JQ, Liu YM, Xie HP, Ye CT. Studies on the effects of the immunosuppressant FK-506 on the high-risk corneal graft rejection. Yan Ke Xue Bao, 2002. 18(3): 160-4.

## Q18 on cyclosporine and tacrolimus eye drops for severe dry eye

**Recommendation 1**

**PICO framework**

Patient: patients with dry eye

Intervention: cyclosporine eye drops

Comparison: artificial tears

Outcome: all outcomes of interest were listed in the eTable below.

### Table S25. Summary of Findings and Certainty of Evidence Using GRADE Approach^1^

| **Quality assessment** | | | | | | | | **Summary of findings** | | | |
| --- | --- | --- | --- | --- | --- | --- | --- | --- | --- | --- | --- |
|  |  |  |  |  |  |  |  | **Number of patients** | | **Effect size (95%CI)** | **Certainty of evidence** |
| **Outcomes** | **Number of included studies** | **Study design** | **Risk of bias** | **Inconsistency** | **Indirectness** | **Imprecision** | **Publication bias** | **Intervention group** | **Control group** |  |  |
| OSDI | 2 | RCT | General | None | None | Serious | General | 58 | 44 | MD = - 4.75 (-6.31, -3.18) | Moderate |
| BUT | 6 | RCT | General | Serious | None | Serious | General | 160 | 173 | MD = 0.94 (0.08, 1.80) | Low |
| CFS | 3 | RCT | General | Moderate | None | Serious | General | 82 | 70 | MD = - 0.72 (-1.28, -0.16) | Low |
| SIt | 5 | RCT | General | Serious | None | Serious | General | 129 | 140 | MD = 0.45 (-2.25, 3.15) | Low |
| Adverse events | 4 | RCT | General | None | None | Serious | General | 20/142 | 1/151 | OR = 7.70 (93.17, 18.68) | Moderate |

Abbreviation: OSDI=Ocular Surface Disease Index; BUT=break-Up Time; CFS=corneal fluorescein staining; SIT=Schirmer I test; RCT=randomized controlled trial.

**Recommendation 2**

**PICO framework**

Patient: patients with dry eye

Intervention: tacrolimus eye drops

Comparison: placebo or artificial tears or cyclosporine

Outcome: all outcomes of interest were listed in the eTable below.

### Table S26. Summary of Findings and Certainty of Evidence Using GRADE-CERQual Approach

| **Outcomes** | **Study design** | **Methodological limitations** | **Coherence** | **Relevance** | **Adequacy** | **Summary of findings** | **CERQual rating** |
| --- | --- | --- | --- | --- | --- | --- | --- |
| Symptom scores^2-4^ | 3 RCTs | Moderate | High | High | Low, involving 2 countries | The improvement effect of tacrolimus combined with sodium hyaluronate was better than sodium hyaluronate alone | Low |
| BUT^2-4^ | 3 RCTs | Moderate | High | High | Low, involving 2 countries |  | Low |
| CFS^2-4^ | 3 RCTs | Moderate | High | High | Low, involving 2 countries |  | Low |
| SIt^2-4^ | 3 RCTs | Moderate | High | High | Low, involving 2 countries |  | Low |

Abbreviation: OSDI=Ocular Surface Disease Index; BUT=break-Up Time; CFS=corneal fluorescein staining; SIT=Schirmer I test; RCT=randomized controlled trial.

**Drug approval status in the United States, the European Union, and China**

**In the United States,** cyclosporine eye drop was approved for the treatment of dry eye in October 2003; there was no tacrolimus eye drops approved for dry eye.

**In the European Union,** cyclosporine eye drop was approved for the treatment of dry eye in April 2018; there was no tacrolimus eye drops approved for dry eye.

**In China,** cyclosporine eye drop was approved for the treatment of dry eye in June 2020; there was no tacrolimus eye drops approved for dry eye.

**Reference:**

1. Tuan HI, Chi SC, Kang YN. An Updated Systematic Review With Meta-Analysis Of Randomized Trials On Topical Cyclosporin A For Dry-Eye Disease. Drug Des Devel Ther. 2020;14:265-274.
2. Tang XY, Xia JH, Wu Q, Du CX. [Evaluation of 0.1% Tacrolimus Eye Drops in the Treatment of Dry Eye Patients Caused by Chronic Graft-Versus-Host Disease]. Chinese Journal of Optometry Ophthalmology and Visual Science. 2020, 22(3): 172-179.
3. Gu ZY, Liao RF. [Efficacy of FK506 in the treatment of dry eye patients with chronic graft-versus-host disease]. Journal of Clinical Ophthalmology. 2017,25(04):289-292.
4. Moawad P, Shamma R, Hassanein D, Ragab G, El Zawahry O. Evaluation of the effect of topical tacrolimus 0.03% versus cyclosporine 0.05% in the treatment of dry eye secondary to Sjogren syndrome. European journal of ophthalmology. 2022 Jan;32(1):673-679.

## Q19 on tacrolimus and cyclosporine eye drops for Mooren ulcer

**Recommendation**

**PICO framework**

Patient: patients with Mooren ulcers

Intervention: tacrolimus or cyclosporine eye drops

Comparison: other immunosuppressants

Outcome: all outcomes of interest were listed in the eTable below.

### Table S27. Summary of Findings and Certainty of Evidence Using GRADE-CERQual Approach

| **Outcomes** | **Study design** | **Methodological limitations** | **Coherence** | **Relevance** | **Adequacy** | **Summary of findings** | **CERQual rating** |
| --- | --- | --- | --- | --- | --- | --- | --- |
| Corneal ulcer healing^1-7^ | 1 cohort study  6 case series | Moderate | High | High | Moderate, involving 2 countries | Tacrolimus and cyclosporine eye drops improved the healing rate of encroaching corneal ulcer | Low |
| Visual acuity^2-5,7^ | 5 case series | Moderate | Low | High | Moderate, involving 2 countries | Tacrolimus and cyclosporine eye drops as postoperative adjunct improved visual acuity | Very low |
| Recurrence^1-2, 4-7^ | 1 cohort study  5 case series | Moderate | High | High | Moderate, involving 2 countries | Tacrolimus and cyclosporine eye drops decreased the recurrence rate of encroaching corneal ulcer | Low |
| Ocular adverse events^1-3, 6-7^ | 1 cohort study  4 case series | High | Low | High | Moderate, involving 2 countries | Tacrolimus and cyclosporine eye drops were safe as postoperative adjuvant | Very low |

**Drug approval status in the United States, the European Union, and China**

None.

**Reference**

1. Zhang T, Zhu TH, Lin BT, Efficacy of cyclosporine A combined with glucocorticoids in the treatment of cannibalizing corneal ulcers. International Journal of Ophthalmology, 2020. 20(02): 286-289.
2. Tandon, R., Chawla, B., Verma, K., Sharma, N., Titiyal, J. S.. Outcome of treatment of mooren ulcer with topical cyclosporine a 2%. Cornea, 2008. 27(8): 859-61.
3. Chen X, Analysis of the clinical effect of 0.1% tacrolimus eye drops in the treatment of refractory immune-related keratopathy. Capital Food and Medicine 2020, Vol. 27, No. 22, pp. 48-49, 2020.
4. Liao RF, Zhu ML, Zhang XF, Chen T. Irregular graft lamellar keratoplasty in the treatment of erosive corneal ulcer. Journal of Clinical Ophthalmology, 2001. 009(001):14-15.
5. Lin H, Hao YY, Ding SY, He J. Experience in the treatment of 23 eyes with refractory eroding corneal ulcer. Journal of Zhengzhou University (Medical Edition), 2002. 37(005): 699-700.
6. Liu MN, Sun XL, Shi WY. Clinical treatment of refractory eroding corneal ulcer with necrotizing scleritis. Chinese Journal of Optometry and Vision Science, 2013. 15(8): 4.
7. Zhang N, Li SX, Zhang LT, Shi WY. Efficacy observation of 0.1% tacrolimus eye drops in the treatment of refractory immune-related keratopathy. Journal of Clinical Ophthalmology, 2017. 25(3): 5.

## Q20 on topical glucocorticoid for adenoviral keratoconjunctivitis

**Recommendation 1**

**PICO framework**

Patient: patients with acute adenoviral keratoconjunctivitis

Intervention: ophthalmic preparation of glucocorticoid

Comparison: artificial tears

Outcome: all outcomes of interest were listed in the eTable below.

### Table S28. Summary of Findings and Certainty of Evidence Using GRADE Approach

| **Quality assessment** | | | | | | | | **Summary of findings** | | | |
| --- | --- | --- | --- | --- | --- | --- | --- | --- | --- | --- | --- |
|  |  |  |  |  |  |  |  | **Number of patients** | | **Effect size (95%CI)** | **Certainty of evidence** |
| **Outcomes** | **Number of included studies** | **Study design** | **Risk of bias** | **Inconsistency** | **Indirectness** | **Imprecision** | **Publication bias** | **Intervention group** | **Control group** |  |  |
| Incidence of subepithelial infiltrates within 1 month^1-3^ | 2 | RCT | None | Serious | None | Serious | None | 17/42 eyes | 18/42 eyes | RR = 0.98 (0.20, 4.67) | Low |
|  | 1 | Cohort study | None | None | None | None | None | 29/92 eyes | 36/64 eyes | RR = 0.56 (0.39, 0.81) | Low |

Abbreviations: RCT=randomized controlled trial; RR=relative risk.

**Recommendation 2**

**PICO framework**

Patient: patients with chronic adenoviral keratoconjunctivitis

Intervention: ophthalmic preparation of glucocorticoid

Comparison: placebo or other positive control drugs

Outcome: all outcomes of interest were listed in the eTable below.

### Table S29. Summary of Findings and Certainty of Evidence Using GRADE Approach

| **Quality assessment** | | | | | | | | **Summary of findings** | | | |
| --- | --- | --- | --- | --- | --- | --- | --- | --- | --- | --- | --- |
|  |  |  |  |  |  |  |  | **Number of patients** | | **Effect size (95%CI)** | **Certainty of evidence** |
| **Outcomes** | **Number of included studies** | **Study design** | **Risk of bias** | **Inconsistency** | **Indirectness** | **Imprecision** | **Publication bias** | **Intervention group** | **Control group** |  |  |
| Rate of complete elimination of corneal subepithelial infiltration at 6 months^4-5^ | 2 | RCT | None | General | None | Serious | None | 56/73 patients | 51/68 patients | RR = 1.07 (0.71, 1.61) | Moderate |
| Recurrence rate of corneal subepithelial infiltration after drug withdrawal at 6 months^4-5^ | 2 | RCT | None | None | None | Serious | None | 13/83 eyes | 6/79 eyes | RR = 2.05 (0.82, 5.12) | Moderate |
| BCVA at 6 months^4-5^ | 2 | RCT | None | None | None | Serious | None | 73 patients | 68 patients | MD = 0.06 (0.02, 0.09) | Moderate |
| IOP at 6 months^4-5^ | 2 | RCT | None | None | None | Serious | None | 73 patients | 68 patients | MD = 1.13 (0.30, 1.97,) | Moderate |
| Treatment was terminated due to drug intolerance^4-5^ | 2 | RCT | None | None | None | Serious | None | 5/73 patients | 7/68 patients | RR = 0.69 (0.16, 2.94) | Moderate |

Abbreviations: BCVA=best corrected visual acuity; IOP= intraocular pressure; RCT=randomized controlled trial; MD=mean difference; RR=relative risk.

**Drug approval status in the United States, the European Union, and China**

None.

**Reference**

1. Kovalyuk N, Kaiserman I, Mimouni M, Cohen O, Levartovsky S, et al. Treatment of adenoviral keratoconjunctivitis with a combination of povidone-iodine 1.0% and dexamethasone 0.1% drops: a clinical prospective controlled randomized study. Acta Ophthalmol.2017;95(8): e686-e692.
2. Laibson PR, Dhiri S, Oconer J, Ortolan G. Corneal infiltrates in epidemic keratoconjunctivitis. Response to double-blind corticosteroid therapy. Arch Ophthalmol.1970;84(1): 36-40.
3. Asena L, Şıngar Özdemir E, Burcu A, Ercan E, Çolak M, et al. Comparison of clinical outcome with different treatment regimens in acute adenoviral keratoconjunctivitis. Eye (Lond) 2017;31: 781‑787.
4. Gouider D, Khallouli A, Maalej A, Yousfi MA, Ksiaa I, et al. Corticosteroids Versus Cyclosporine for Subepithelial Infiltrates Secondary to Epidemic Keratoconjunctivitis: A Prospective Randomized Double-Blind Study. Cornea. 2021;40(6):726-732.
5. Bhargava R, Kumar P. Comparison of the safety and efficacy of topical Tacrolimus (0.03%) versus dexamethasone (0.05%) for subepithelial infiltrates after adenoviral conjunctivitis. Indian J Ophthalmol.2019;67(5): 594-598.

## Q21 on intravitreal injection of ceftazidime for endophthalmitis

**Recommendation**

**PICO framework**

Patient: patients with infectious endophthalmitis

Intervention: intravitreal injection of ceftazidime combined with other treatments

Comparison: vitrectomy or other drug control treatment

Outcome: all outcomes of interest were listed in the eTable below.

### Table S30. Summary of Findings and Certainty of Evidence Using GRADE-CERQual Approach

| **Outcomes** | **Study design** | **Methodological limitations** | **Coherence** | **Relevance** | **Adequacy** | **Summary of findings** | **CERQual rating** |
| --- | --- | --- | --- | --- | --- | --- | --- |
| Effective rate* | 1 quasi-experimental study plus 4 case series | Moderate | High | Moderate | Moderate, involving three countries | Except for one case series study with adverse outcome, the remaining 4 studies showed the efficacy of intravitreal injection of ceftazidime in the treatment of endophthalmitis. | Very low |

* Inconsistent definition of outcome

**Drug approval status in the United States, the European Union, and China**

None.

**Reference**

1. Narsani AK, Jatoi SM, Gul S, Dabir SA. Efficacy of intravitreal ceftazidime in acute postoperative endophthalmitis.Journal of the College of Physicians and Surgeons Pakistan.2008;18 (2): 98-101.
2. Dave VP, Pathengay A, Behera S, Joseph J,Sharma S,Pappuru RR,Das T. Enterobacter endophthalmitis: clinical settings, susceptibility profile, and management outcomes across two decades. Indian Journal of Ophthalmol. 2019;68(1):112-117.
3. Lohano MK, Kamal M, Junejo SA. Visual acuity outcomes after intra vitreal ceftazidime in acute post-operative endophthalmitis. Pakistan Journal of Medical Sciences. 2012;(28) 3:471-475.
4. Gong L, Jiang DY.[Analysis of clinical effect on post-traumatic endophthalmitis in children].International Eye Science.2015;15(6):1040-1042.
5. Yuan RD,He XG,Liu SZ, Wang WG.[Effect of vitrectomy combined with intraocular injection on traumatic endophthalmitis]. Journal of traumatic surgery.2007;9(3):234-236.

## Q22 on intravitreal injection of amikacin for endophthalmitis

**Recommendation**

**PICO framework**

Patient: patients with endophthalmitis, both exogenous and endogenous

Intervention: intravitreal injection of amikacin combined with other treatments

Outcome: all outcomes of interest were listed in the eTable below

### Table S31. Summary of Findings and Certainty of Evidence Using GRADE-CERQual Approach

| **Outcomes** | **Study design** | **Methodological limitations** | **Coherence** | **Relevance** | **Adequacy** | **Summary of findings** | **CERQual rating** |
| --- | --- | --- | --- | --- | --- | --- | --- |
| Visual acuity^1-5^ | 5 case series | High | Moderate | High | Low, involving four countries | Intravitreal injection of amikacin for endophthalmitis might improve visual acuity. | Very low |

Abbreviations: BCVA=best corrected visual acuity.

**Drug approval status in the United States, the European Union, and China**

None.

**Reference**

1. Bajimaya S, Kansakar I, Sharma BR, Byanju R. Outcome of cluster endophthalmitis in western plain region of Nepal. Kathmandu University Medical Journal. 2010 Mar;8(29):102-8.
2. Liu W, Ye B, Huang H, Li Ch, Lan LX. [Clinical analysis of vitrectomy combined with silicone oil filling for traumatic endophthalmitis]. Jiangxi western medicine. 2011;46(10):937-939.
3. Okhravi N, Towler HM, Hykin P, Matheson M, Lightman S. Assessment of a standard treatment protocol on visual outcome following presumed bacterial endophthalmitis. British Journal of Ophthalmology. 1997 Sep;81(9):719-25.
4. Wade PD, Khan SS, Khan MD. Endophthalmitis: magnitude, treatment and visual outcome in northwest frontier province of Pakistan. Annals of African Medicine. 2009 Jan-Mar;8(1):19-24.
5. Gautam P, Joshi SN, Sharma A, Thapa M, Shah DN, et al. Outcome of the patients with post-operative cluster endophthalmitis referred to a tertiary level eye care center in Nepal. Nepalese Journal of Ophthalmology. 2013 Jul-Dec;5(2):235-41.

## Q23 on intravitreal injection of vancomycin for endophthalmitis

**Recommendation**

**PICO framework**

Patient: patients with endophthalmitis, both exogenous and endogenous

Intervention: intravitreal injection of vancomycin combined with other treatments

Comparison: non-vitreous injection of vancomycin combined with other treatments

Outcome: all outcomes of interest were listed in the eTable below.

### Table S32. Summary of Findings and Certainty of Evidence Using GRADE-CERQual Approach

| **Outcomes** | **Study design** | **Methodological limitations** | **Coherence** | **Relevance** | **Adequacy** | **Summary of findings** | **CERQual rating** |
| --- | --- | --- | --- | --- | --- | --- | --- |
| Effective rate*^1^ | 1 cohort study | High | Not applicable | High | Low | The effective rate of vancomycin intravitreal injection was significantly different from that of gentamicin intravitreal injection (50% vs. 16.67%, P<0.05). | Very low |
| Visual acuity^2-5^ | 4 case series | High | High | Moderate | Moderate, 4 studies were all from the United States | Vitreous injection of vancomycin in the treatment of bacterial exogenous endophthalmitis improved visual acuity significantly after intervention. | Very low |

* Inconsistent definition of outcome

**Drug approval status in the United States, the European Union, and China**

None.

**Reference**

1. Zhou J, Chen QY, Zhou XT. [Clinical observation of intravitreal vancomycin injection in the treatment of ophthalmitis] . Journal of Clinical Ophthalmology. 1998(05)：310-311.
2. Yannuzzi NA, Si N, Relhan N, Kuriyan AE, Albini TA, et al. Endophthalmitis After Clear Corneal Cataract Surgery: Outcomes Over Two Decades. American Journal of Ophthalmol. 2017 Feb;174:155-159.
3. Yannuzzi NA, Patel NA, Relhan N, Tran KD, Si N, et al. Clinical Features, Antibiotic Susceptibilities, and Treatment Outcomes of Endophthalmitis Caused by Staphylococcus epidermidis. Ophthalmol Retina. 2018 May;2(5):396-400.
4. Lalwani GA, Flynn HW Jr, Scott IU, Quinn CM, Berrocal AM, et al. Acute-onset endophthalmitis after clear corneal cataract surgery (1996-2005). Clinical features, causative organisms, and visual acuity outcomes. Ophthalmology. 2008 Mar;115(3):473-6.
5. Kuriyan AE, Weiss KD, Flynn HW Jr, Smiddy WE, Berrocal AM, et al. Endophthalmitis caused by streptococcal species: clinical settings, microbiology, management, and outcomes. American Journal of Ophthalmol. 2014 Apr;157(4):774-780.

## Q24 on intravitreal injection of voriconazole for fungal endophthalmitis

**Recommendation**

**PICO framework**

Patient: patients with fungal endophthalmitis

Intervention: intravitreal injection of voriconazole

Comparison: non-vitreous injection of voriconazole combined with other treatments

Outcome: all outcomes of interest were listed in the eTable below.

### Table S33. Summary of Findings and Certainty of Evidence Using GRADE-CERQual Approach

| **Outcomes** | **Study design** | **Methodological limitations** | **Coherence** | **Relevance** | **Adequacy** | **Summary of findings** | **CERQual rating** |
| --- | --- | --- | --- | --- | --- | --- | --- |
| Effective rate*^1-6^ | 1 RCT  5 case series | Low | High | Moderate | Moderate, involving five countries | Except for one case series study, the other five studies showed that intravitreal injection of voriconazole was effective in the treatment of endophthalmitis. | Very low |

* Inconsistent definition of outcome

**Drug approval status in the United States, the European Union, and China**

None.

**Reference**

1. Li XD. [Intravitreal injection of drugs combined with vitrectomy on fungal endophthalmitis]. International Eye Science. 2017;17(3):511-515.
2. Sen S, Lalitha P, Mishra C, Parida H, Rameshkumar G, et al. Post-cataract Surgery Fungal Endophthalmitis: Management Outcomes and Prognostic Factors[J]. Ocular Immunology Inflammation. 2020;2(17):1-8.
3. Thomas PM，Joseph P. Candida dubliniensis endophthalmitis: five cases over 15 years. Journal of Ophthalmic Inflammation and Infection. 2013; 3(1):66-72.
4. Mithal K, Pathengay A, Bawdekar A, Jindal A, Vira D, et al. Filamentous fungal endophthalmitis: results of combination therapy with intravitreal amphotericin B and voriconazole. Clinical ophthalmology. 2015;9(1):649-655.
5. Vasconcelos-Santos DV, Nehemy MB . Use of voriconazole in the surgical management of chronic postoperative fungal endophthalmitis. Ophthalmic Surgery Lasers & Imaging the Official Journal of the International Society for Imaging in the Eye. 2009;40(4):425-431.
6. Bui DK, Carvounis P. Favorable Outcomes of Filamentous Fungal Endophthalmitis Following Aggressive Management. Journal of ocular pharmacology and therapeutics: The official journal of the Association for Ocular Pharmacology and Therapeutics, 2016;0(0):1-8.

## Q25 on intravitreal injection of amphotericin B for fungal endophthalmitis

**Recommendation**

**PICO framework**

Patient: patients with fungal endophthalmitis

Intervention: intravitreal injection of amphotericin B

Comparison: non-vitreous injection of amphotericin B combined with other treatments

Outcome: all outcomes of interest were listed in the eTable below.

### Table S34. Summary of Findings and Certainty of Evidence Using GRADE-CERQual Approach

| **Outcomes** | **Study design** | **Methodological limitations** | **Coherence** | **Relevance** | **Adequacy** | **Summary of findings** | **CERQual rating** |
| --- | --- | --- | --- | --- | --- | --- | --- |
| Effective rate*^1-13^ | 1 RCT  12 case series | Moderate | High | Moderate | Moderate, involving four countries | Thirteen studies showed that intravitreal amphotericin B was effective in the treatment of endophthalmitis. | Very low |

* Inconsistent definition of outcome

**Drug approval status in the United States, the European Union, and China**

None.

**Reference**

1. Li XD. [Intravitreal injection of drugs combined with vitrectomy on fungal endophthalmitis]. International Eye Science. 2017;17(3):511-515.
2. Thomas PM，Joseph P. Candida dubliniensis endophthalmitis: five cases over 15 years. Journal of Ophthalmic Inflammation and Infection. 2013; 3(1):66-72.
3. Roy DB, Harry WF, John GC, Stephen CP, William W , et al. Endogenous Candida endophthalmitis. Management without intravenous amphotericin B. Ophthalmology. 1990;97(5):666-674.
4. Mithal K, Pathengay A, Bawdekar A, Jindal A, Vira D, et al. Filamentous fungal endophthalmitis: results of combination therapy with intravitreal amphotericin B and voriconazole. Clinical ophthalmology. 2015;9(1):649-655.
5. Chakrabarti A, Shivaprakash MR, Singh R, Tarai B, George VK, et al. Fungal endophthalmitis: fourteen years' experience from a center in India. Retina.2008;28(10):1400-1408.
6. Majji A B, Jalali S, Das T, Gopinathan U. Role of intravitreal dexamethasone in exogenous fungal endophthalmitis. Eye.1999;13(5):660-666.
7. Walter HS，Eddy T，Richard AJ，Vincent GP，Robert DS, etal. Epidemic Postsurgical Candida Parapsilosis Endophthalmitis: Clinical Findings and Management of 15 Consecutive Cases. Ophthalmology.1985;92 (2):1701-1709.
8. Pflugfelder SC, Flynn HW, Zwickey TA, Forster RK, Tsiligianni A, et al. Exogenous Fungal Endophthalmitis. Ophthalmology.1988;95(1):19-30.
9. Ren YG, Zhao YY, Li M. [Clinical diagnosis and treatment of fungal endophthalmitis in 12 patients. Journal of Clinical Ophthalmology]. 2020, 28(1):42-44.
10. Narang S, Gupta A, Gupta V, Dogra MR, Ram J, et al. Fungal endophthalmitis following cataract surgery: clinical presentation, microbiological spectrum, and outcome[J]. American Journal of Ophthalmology. 2001;132(5):609-617.
11. Weishaar PD , Flynn HW , Murray TG, Davis JL, Barr CC, et al. Endogenous Aspergillus endophthalmitis. Clinical features and treatment outcomes. Ophthalmology. 1998;105(1):57-65.
12. Ghoraba HH, Ellakwa AF, Elgemai EM, Mansour HO, Heikal MA. Results of pars plana vitrectomy for the management of endogenous fungal endophthalmitis after urinary tract procedures. Retinal Cases & Brief Reports. 2017;11(2):171-177.
13. Gao S, Gao W, Zhao YL. [Treatment of vitrectomy combined with Liposomal amphotericin B intraocular injection for fungal endophthalmitis]. International Journal Ophthalmol. 2008;8(9):1932-1934.
